# Synthesis, Biological Assessment, and Structure Activity Relationship Studies of New Flavanones Embodying Chromene Moieties

**DOI:** 10.3390/molecules25030544

**Published:** 2020-01-27

**Authors:** Eman Assirey, Azhaar Alsaggaf, Arshi Naqvi, Ziad Moussa, Rawda M. Okasha, Tarek H. Afifi, Alaa S. Abd-El-Aziz

**Affiliations:** 1 Department of Chemistry, Taibah University, Madinah 30002, Saudi Arabia; eman_assirey@hotmail.com (E.A.); aalsaggaf@upei.ca (A.A.); arshi_84@yahoo.com (A.N.); rawdao@yahoo.com (R.M.O.);; 2 Department of Chemistry, University of Prince Edward Island, 550 University Avenue, Charlottetown, PEI C1A 4P3, Canada; 3 Department of Chemistry, College of Science, United Arab Emirates University, Al Ain 15551, UAE; zmoussa@uaeu.ac.ae

**Keywords:** flavanone-containing chromene motifs, in-silico studies, antimicrobial examination, cytotoxic behavior, molecular modeling, SAR analysis

## Abstract

Novel flavanones that incorporate chromene motifs are synthesized via a one-step multicomponent reaction. The structures of the new chromenes are elucidated by using IR, ^1^H-NMR, ^13^C-NMR, ^1^H-^1^H COSY, HSQC, HMBC, and elemental analysis. The new compounds are screened for their in vitro antimicrobial and cytotoxic activities. The antimicrobial properties are investigated and established against seven human pathogens, employing the agar well diffusion method and the minimum inhibitory concentrations. A majority of the assessed derivatives are found to exhibit significant antimicrobial activities against most bacterial strains, in comparison to standard reference drugs. Moreover, their cytotoxicity is appraised against four different human carcinoma cell lines: human colon carcinoma (HCT-116), human hepatocellular carcinoma (HepG-2), human breast adenocarcinoma (MCF-7), and adenocarcinoma human alveolar basal epithelial cell (A-549). All the desired compounds are subjected to in-silico studies, forecasting their drug likeness, bioactivity, and the absorption, distribution, metabolism, and excretion (ADME) properties prior to their synthetic assembly. The in-silico molecular docking evaluation of all the targeted derivatives is undertaken on gyrase B and the cyclin-dependent kinase. The in-silico predicted outcomes were endorsed by the in vitro studies.

## 1. Introduction

Computational techniques perform a crucial role in many drug discovery programs, from hit identification to the optimization of the lead and thereafter. Numerous computational tools are accessible, and these are categorized as either ligand-based or receptor-based. Screening chemical compounds allows for the elimination of searching an entire chemical space by utilizing libraries of specific, accessible molecules. This not only prevents unnecessary syntheses but also limits the number of derivatives with biologically-compelling behaviors. Sieves may be employed to make certain that the archive satisfies the criteria of biological significance or ‘drug-likeness.’ General sieving strategies for ‘drug-likeness’ [1], which are applicable for the compound databases, are based on Lipinski’s rule-of-five [2], which comprises a series of guidelines centered on hydrophobicity, lipophilicity, and molecular weight and yields a straightforward profile for orally bioavailable molecules. Additional physical sieves could involve a constraint on the polar surface area or the quantity of rotatable bonds of the compounds [3].

The undesirable pharmacokinetic attributes of potential drugs impose restrictions in constructing a headway in the process of novel drug developments; hence, there is an amplified urge to develop new techniques that are intelligent enough to predict these drug traits [4,5,6]. The scrutiny of a drug’s pharmacokinetic features comprises absorption, distribution, metabolism, and excretion (ADME) [7]. The ADME guidelines dictate whether drug candidates are to be proceeded with, clasped, or ceased [8].

The cyclin-dependent kinases (CDK/cyclins) constitute a taxon of heterodimeric kinases that perform vital functions in the modulation of the cell cycle progression, transcription and other crucial biological affairs, such as neuronal metabolism and differentiation [9]. The constitutive or deregulated hyperactivity of these kinases as a consequence of the overexpression, amplification, or mutation of cyclins or CDK generates the proliferation of cancer cells, and the peculiar task of the preceding kinases has been implicated in a broad assortment of human cancers [10]. Therefore, these kinases represent the biomarkers of proliferation and arrest with the pharmacological intent of the development of anticancer therapeutics [11]. The overexpression of the cyclin-dependent kinase-2 (CDK2) has been documented in various categories of human tumors [12,13,14,15,16,17]. Molecular docking evaluations have been executed to acquire an additional comprehension of the binding behavior of the generated molecules with CDK2 (Protein Data Bank (PDB) ID: 1FVV) as well as gyrase B (GyrB43) from *Escherichia coli* (PDB code: 4PRV), which has been reported to be a target for analyzing antibacterial activities [18].

As a continuation of our preceding work in the area of the synthesis and biological screening of the bioactive molecules [19,20,21,22] based on extensive molecular modeling and in-silico studies, we carried out the synthesis of a novel series of chromene-incorporating flavanones, endeavoring to find new and alternative drug candidates to replace those in peril of facing resistance from microorganisms.

The substantial upsurge of microorganisms’ resistance to antimicrobial agents is one of the foremost concerns among scientists and clinicians worldwide. Moreover, numerous pathogenic viruses, bacteria, fungi, and protozoa have subsequently developed resistance, which renders treatment much more challenging with the existing drugs [23]. Furthermore, the abuse of synthetic antibiotics has contributed to an increased incidence of bacterial resistance to available antibacterial agents, ensuing an urgent need for natural antimicrobials [24]. To overcome the aforementioned drawbacks of the modern antimicrobial drugs and to attain more efficacious drugs, antimicrobial agents encompassing a novel mode of action should be acquired [25].

**Figure 1:**
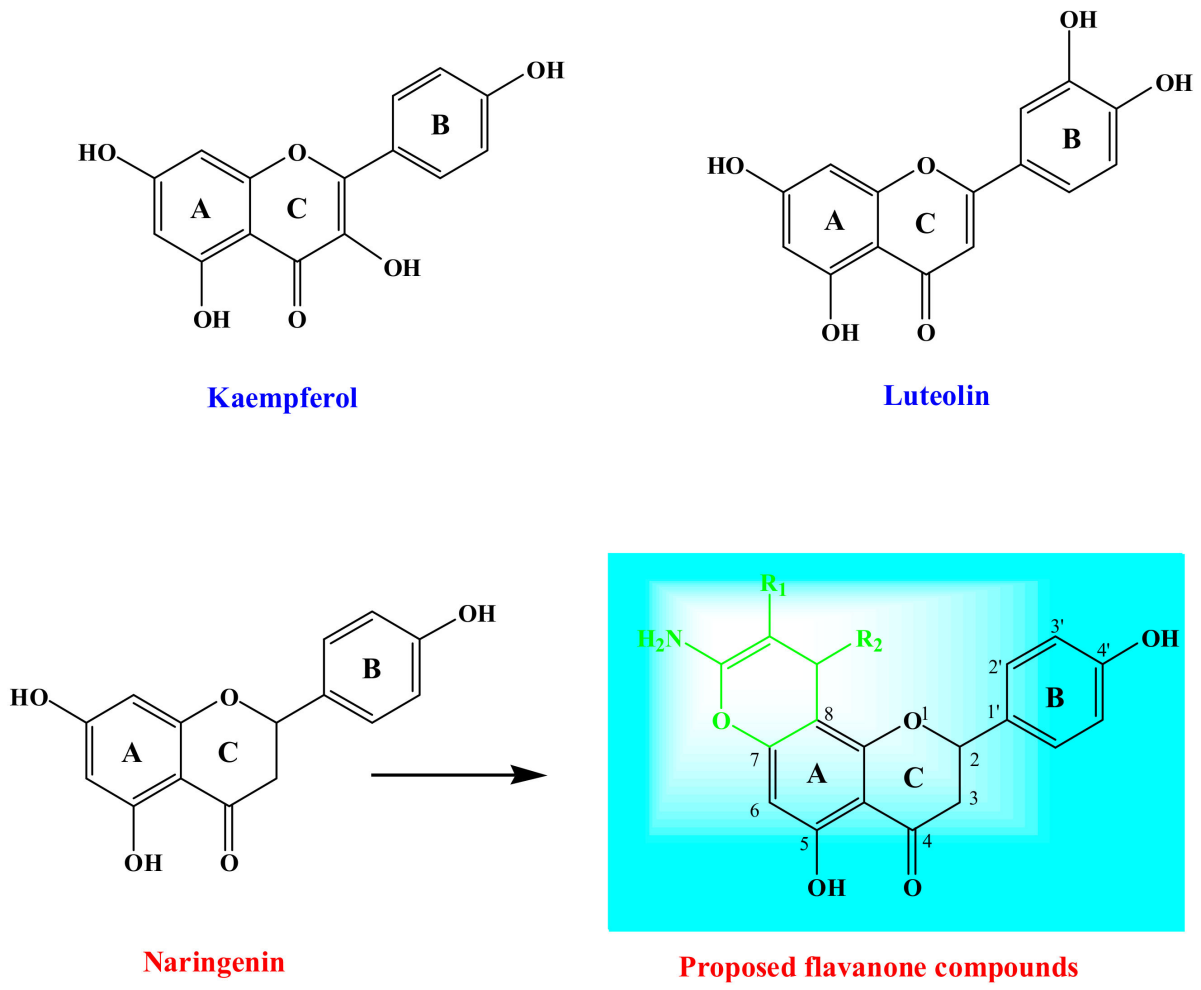
The design of a novel flavanone linked to chromene moiety.

Plant-derived flavonoids are a class of naturally-occurring phenyl chromenes that are widely-distributed in edible plants and have been observed in fruits, vegetables, tea, and wine [26,27]. These natural products possess a range of biological activities, including those of the anti-inflammatory, antiallergic, antiproliferative, antibacterial, antidiabetic, antiviral, antimutagenic, antithrombotic, anticarcinogenic, estrogenic, hepatoprotective, insecticidal, and antioxidant varieties [28,29]. Figure 1 illustrates two examples of the flavanone family that are used for cancer resistance treatment (Kaempferol) and for treating inflammatory disorders (Luteolin). The antibacterial activities of flavonoids have been reported to be correlated with their chemical structures [29,30,31,32]. However, the quantitative structure–activity relationship (QSAR) for flavonoids as antibacterial agents has been capturing interest through the quantitative construction of associations between the molecular structures or properties with a variation in biological activities [18,33]. It is significant that anticancer activity, which is comprised of the most interesting pharmacological properties of flavonoids [34], proceeds via a unique mechanism, and aids in the prevention of cancer growth through the flavonoids’ ability to function as anti-oxidants [35,36,37], enzyme inhibitors [38], and growth regulators [39]. Moreover, the biological performance of flavonoid molecules is reliant on their position and number of substitutions as well as their structures’ condensation level, namely glucosides, homodimers, heterodimers, hydroxy groups, and isoprenyl units [40,41].

Previous studies have conveyed that a methoxy or hydroxy substituent at the C-7 position of the flavanone enhances inhibitory effects on the human colon carcinoma (HCT)-116 cell line [42]. The established results directed our attempt to explore the design of flavanone derivatives with bulkier substituents at the C-7 position and to elucidate their inhibitory effect. Even though several flavonoid derivatives, modified at the C-7 position, have been reported, Naringenin (Figure 1), 4′,5,7-trihydroxyflavanone derivatives, functionalized at the C-7 position, have seldom been investigated [43,44]. Naringenin functions as an essential chemical species that operates as an estrogenic substance in humans and as an endogenous regulator in plants [45]. Based on these considerations, herein, we report the synthesis of chromene-based 4′,5,7-trihydroxyflavanone compounds (Figure 1) and explore their antimicrobial and antiproliferative behaviors, as advocated by the theoretical predictions. The characterization of the proposed molecules was done through in-silico studies, forecasting their drug likeness, bioactivity, and ADME properties. In addition, the Naringenin derivatives Kaempferol and Luteolin exhibited distinguished biological activities. Kaempferol reduces the resistance of cancer cells to anti-cancer drugs, such as Vinblastine and Paclitaxel, while Luteolin has been employed as a Chinese traditional medicine for treating hypertension, inflammatory disorders, and cancer.

## 2. Results and Discussion

### 2.1. Synthesis and Characterization 

**Scheme 1:**
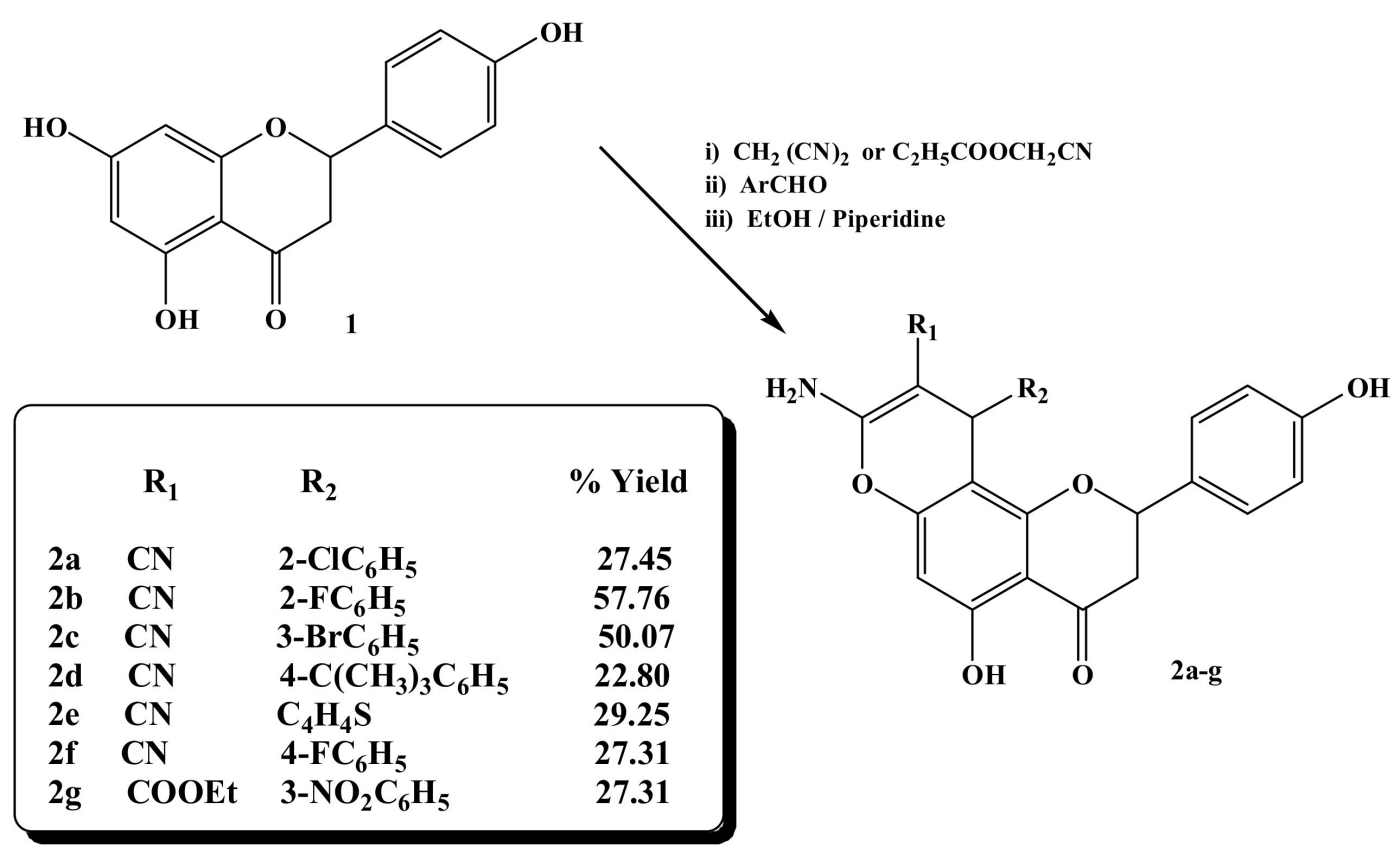
Synthesis of 8-amino-10-phenyl-5-hydroxy-2-(4-hydroxyphenyl)-4-oxo-3,4-diydro-2*H*,10*H*-pyrano [2, 3-f] chromene derivatives **2a**–**2g**.

The target compounds **2a**–**2g** were synthesized, as outlined in Scheme 1. The condensation reaction between the 4′,5,7-trihydroxyflavanone, malononitrile or ethyl cyanoacetate, and the aromatic aldehydes in the presence of piperidine in refluxing ethanol yielded the chromene derivatives **2a**–**2g**, yield (22.8%–57.8%).

**Scheme 2:**
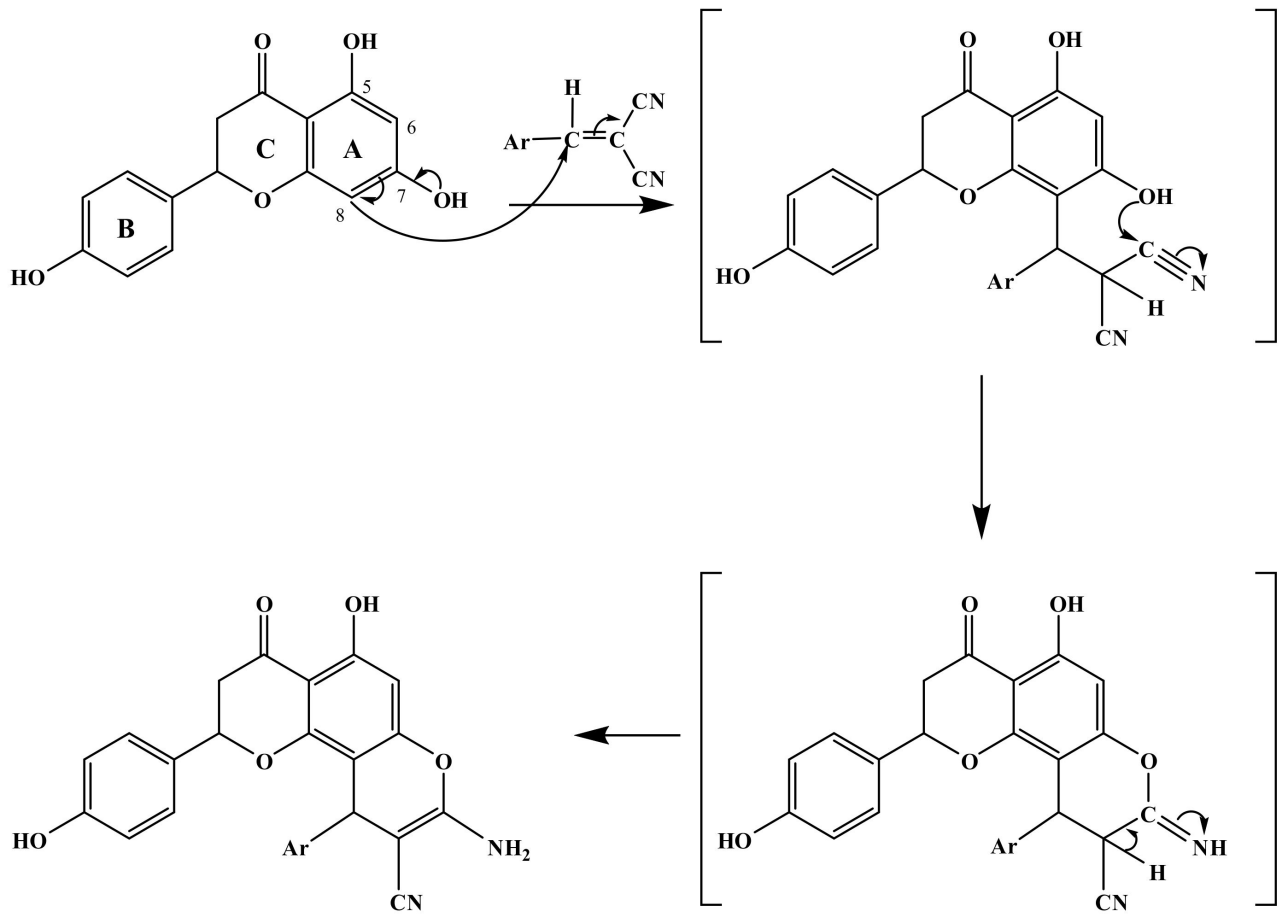
Mechanistic pathway leading to **2a**–**2g**.

The first step of the multicomponent coupling process began with a Knoevenagel condensation between malononitrile or ethyl cyanoacetate, and the aromatic aldehydes, followed by a Michael addition of the C-8 carbon of 4′,5,7-trihydroxyflavanone to the α,β-unsaturated malononitrile intermediate, re-aromatization, intramolecular cyclization involving the addition of the C-7 hydroxyl group of 4′,5,7-trihydroxyflavanone onto one of the cyano groups, and subsequent tautomerization [46], as shown in Scheme 2.

Although both the ortho (C6) and para (C8) positions (relative to the C-5 hydroxyl group) of 5,7-dihydroxy-2-(4-hydroxyphenyl) chroman-4-one are strongly and almost as equally activated towards the electrophilic attack, only the C-8 position reacted. The ^13^C chemical shifts of the C-6 and C-8 carbons are 96.7 (C_6_) and 95.8 (C_8_) ppm, respectively, which indicates that they are in similar electronic environment and exhibit similar nucleophilicity. This suggests that the regiochemistry depended on steric factors to a much larger extent than electronic factors. Indeed, the C-8 position is much less hindered, especially considering the six-membered ring that may form due to hydrogen bonding between the C5–OH and the C-4 carbonyl group. Thus, a preferential attack at C-8 was observed. It is also noteworthy that the C-3′ of the phenol ring did not show any reactivity under the reaction conditions since it is much less activated than C-6 and C-8. The observed downfield chemical shift of C-3′ (116.0 ppm for C-3′ compared to 96.7 ppm for C-6 and 95.8 ppm for C-8) clearly suggests that C-3′ experiences less delocalization of electron density from the lone phenolic OH, rendering the C-3′ position the least nucleophilic site compared to C-6 and C-8 positions.

**Figure 2:**
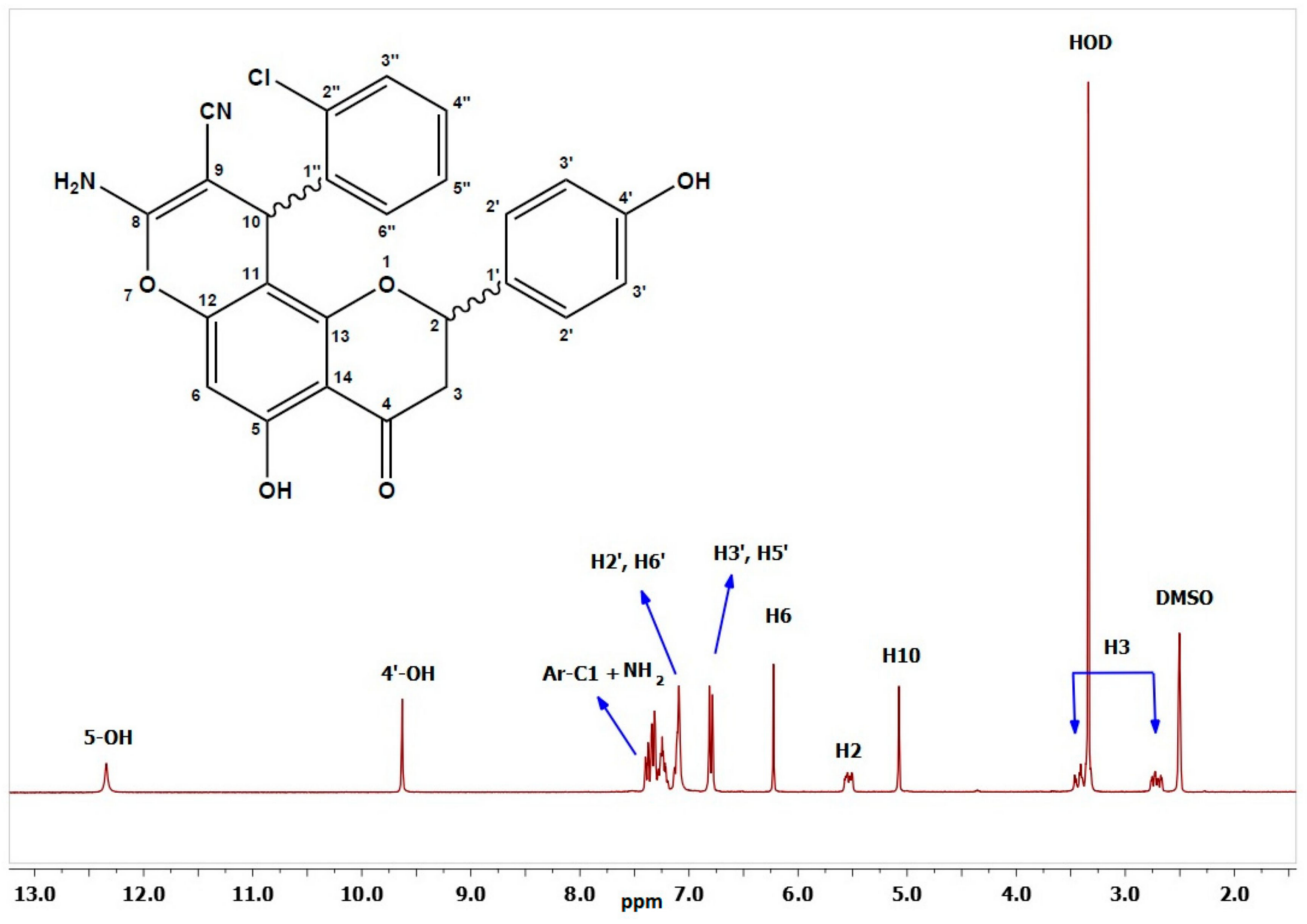
^1^H-NMR of Compound **2a.**

The structures of the newly synthesized derivatives were confirmed by various spectroscopic and analytical techniques, such as FT-IR, ^1^H-NMR, ^13^C-NMR, COSY, HMBC, HSQC, and elemental analysis. The FT-IR spectra exhibited characteristic absorption bands between 2178 and 2207 for the CN group, while the NH_2_ stretching were in the range of 3381–3424 cm^−1^. Moreover, the C=O group displayed bands at 1634–1686 cm^−1^, and the OH bands appeared as broad absorptions at 3477–3634 cm^−1^. The ^1^H-NMR spectra of **2a**–**2g**, were measured in dimethyl sulfoxide (DMSO-*d*_6_) where the H2 signal appeared as a doublet of doublets in the range of 5.07–5.64 ppm. The resonances of the diastereotopic H3ax and H3eq protons 3.34–3.51 and 2.72–2.83 ppm, respectively, as two doublets of doublets. The H3ax and H3eq of the chromene ring were coupled with a constant of 17.1–17.6 Hz. The value of the coupling constant between H2 and H3ax (*J* = 12.2–13.5 Hz) indicates a *trans*-diaxial coupling. Thus, the H2 was axial, and the aryl group linked to the H2 had equatorial orientation. As anticipated, the hydroxyl group attached to the C5 appeared as a singlet at 11.9–12.5 ppm, and the C4′–OH occurred in the range 9.62–9.63 ppm; meanwhile, the pyran H10 methine exhibited chemical shifts at 4.46–5.08 ppm, and the amine protons resonated at 7.03–7.79 ppm. The singlet at 6.21–6.27 ppm corresponded to H6, while the remaining aromatic protons resonated further downfield between 6.74 and 8.08 ppm. As an example, the proton spectrum of **2a** is shown in Figure 2. In the proton spectrum of Compound **2g**, the methyl protons appeared at 0.98 ppm as a triplet, while the methylene chemical shift of the ethoxy group OCH_2_CH_3_ ranged from 3.81 to 4.03 ppm.

**Figure 3:**
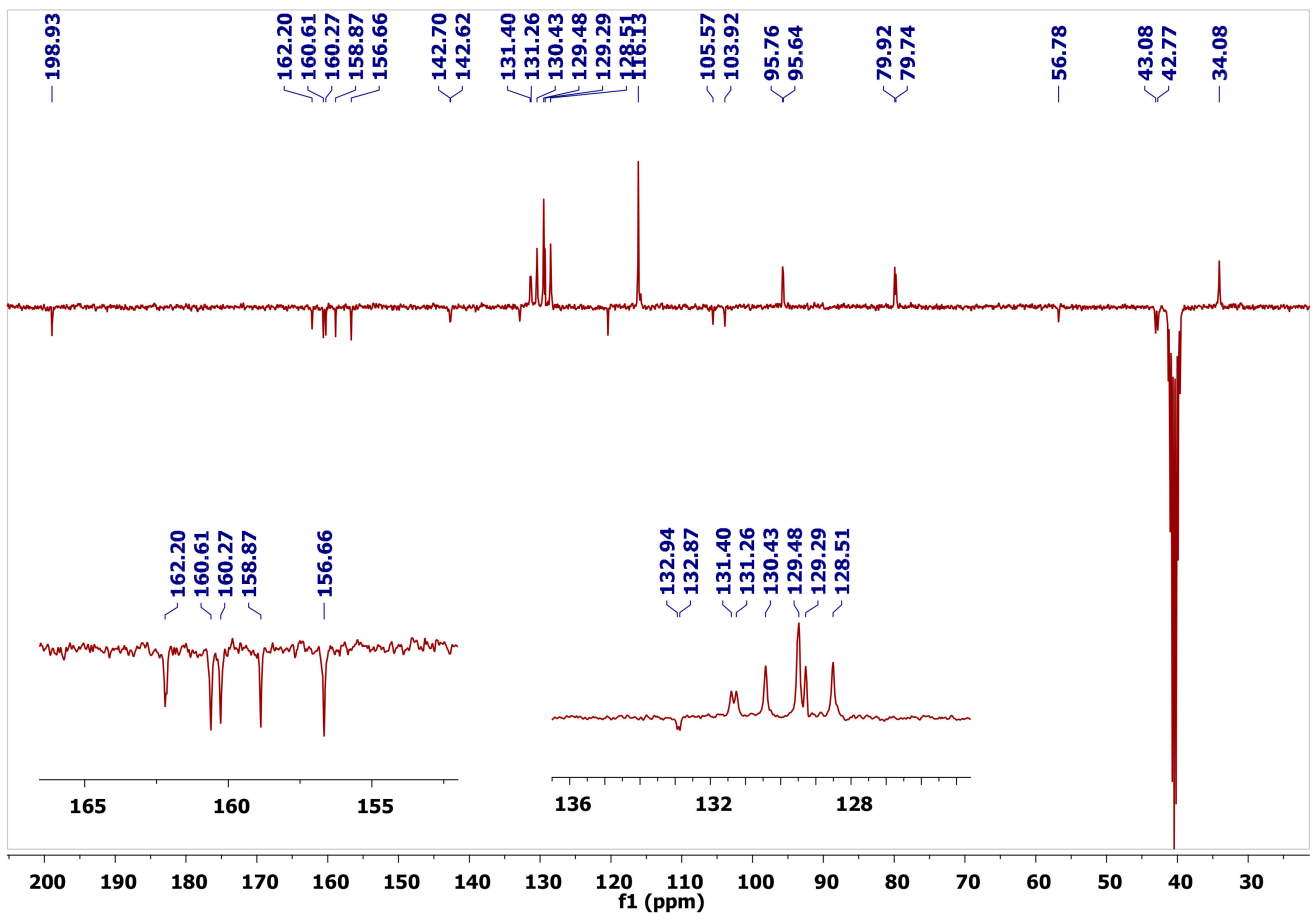
Distorsionless Enhancement by Polarization Transfer Including the Detection of Quaternary Nuclei (DEPTQ-135) ^13^C-NMR of Compound **2a**.

The ^13^C-NMR spectra of the new molecules exhibited signals ranging from 78.8–80.2 ppm and 41.7–43.4 ppm that corresponded to C-2 and C-3, respectively, where the lower field signal was being assigned to the oxygenated C-2 carbon. The C-6 signal resonated at 94.8–96.8 ppm, whereas the B ring carbons C-2′, and C-6′ showed up at 128.5–129.5 ppm with the equivalent C-3′ and C-5′ appearing at 115.9–116.1 ppm. The C-4′ carbon, connected to the hydroxyl group, appeared at 157.9–158.9 ppm, and the signal at 128.4–132.8 ppm was ascribed to C-1′. The signal at 30.3–36.2 ppm corresponded to the C-10 of the pyran ring, while the quaternary carbon C-8 attached to amine group appeared in the range of 159.3–161.9 ppm. The cyanide functionality, which was a distinctive signal, resonated further downfield at 119.7–121.0 ppm, while the aromatic carbons showed signals between 162.9 and 102.8 ppm, with an additional signal for the C=O of the flavanone moiety at 198.0–198.9 ppm (Figure 3). The carbon signal for the alkoxy methyl group of **2g** resonated at 15.0 ppm, the methylene carbon of -OCH_2_CH_3_ had a chemical shift at 59.8 ppm, and the signal for (C=O ester) emerged at 168.7 ppm.

### 2.2. Computational Studies

The proposed molecules of flavanone-containing chromenes were evaluated for their in-silico bioactivity, physicochemical, pharmacokinetic/ADME, and drug likeness traits. The bioactivity scores were forecasted against six different preset protein structures within the Molinspiration software, namely the G Protein Coupled Receptors (GPCR) ligand, the ion channel modulator, the kinase inhibitor, the nuclear receptor ligand, and the protease and enzyme inhibition. The results are documented in Table 1. The obtained values indicated the binding affinity of the assessed compounds, **2a**–**2g**, to the aforementioned enzymes and receptors, where the positive values suggested a greater affinity and the negative values implied a lower one. All the tested compounds showed low bioactivity towards the selected protein structures except for the nuclear receptor ligand.

Furthermore, the assorted physicochemical parameters like the number of rotatable bonds, the count of specific atom class, molar refractivity, lipophilicity, and water solubility were calculated. A very effective physiochemical variable, i.e., TPSA (topological polar surface area) was appraised for assessing the drug transport attributes. These physicochemical properties are tabulated in Table 2.

**Figure 4:**
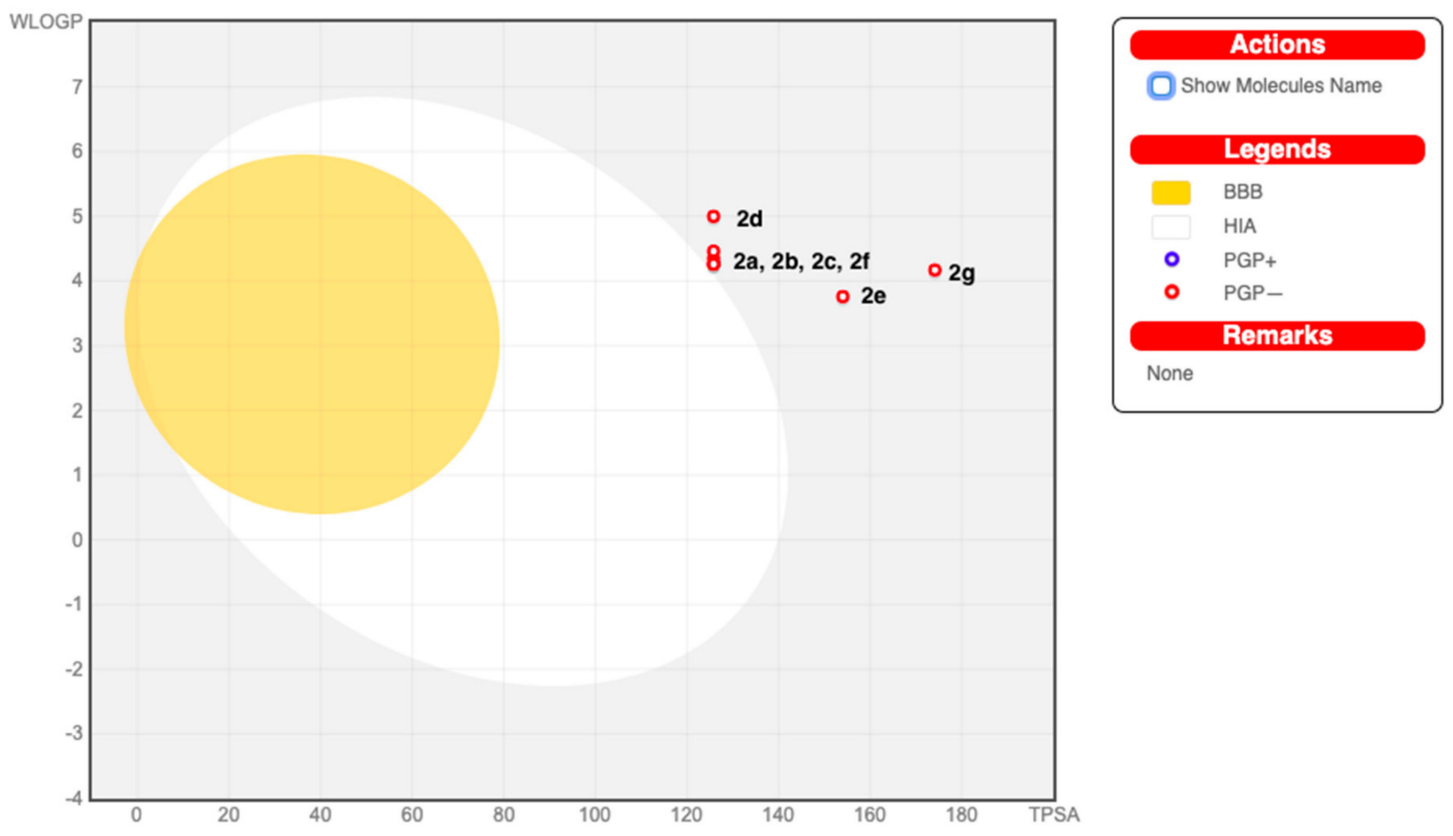
BOILED-Egg diagram of Compounds **2a**–**2g**. (BBB=Blood brain barrier, HIA= human gastrointestinal absorption, PGP+ = P-glycoprotein substrate inhibitor, PGP- = P-glycoprotein substrate non-inhibitor).

The predicted pharmacokinetic/ADME properties of the new series, **2a**–**2g**, are given in Table 3. All of the examined molecules exhibited no gastrointestinal (GI) absorption and were P-gp (p-glycoprotein) non-inhibitors. None of these molecules were able to permeate through the blood–brain barrier (BBB). The forecast for the passive HIA (human gastrointestinal absorption), the BBB permeations, and the P-gp substrates was clubbed in an intuitive graphical classification model, namely the BOILED-Egg diagram as shown in Figure 4. Meanwhile, the examined series inhibited the cytochrome P450 isomers, i.e., CYP2C19, CYP2C9, and CYP3A4—except for Compound **2g**, which did not inhibit CYP2C19. On the other hand, all the screened molecules emerged as non-inhibitors of the cytochromes CYP1A2 and CYP2D6. The skin permeability coefficient (log Kp; with Kp in cm/s) values that were evaluated for the flavanone/chromene compounds were discovered to be low skin permeable.

**Figure 5:**
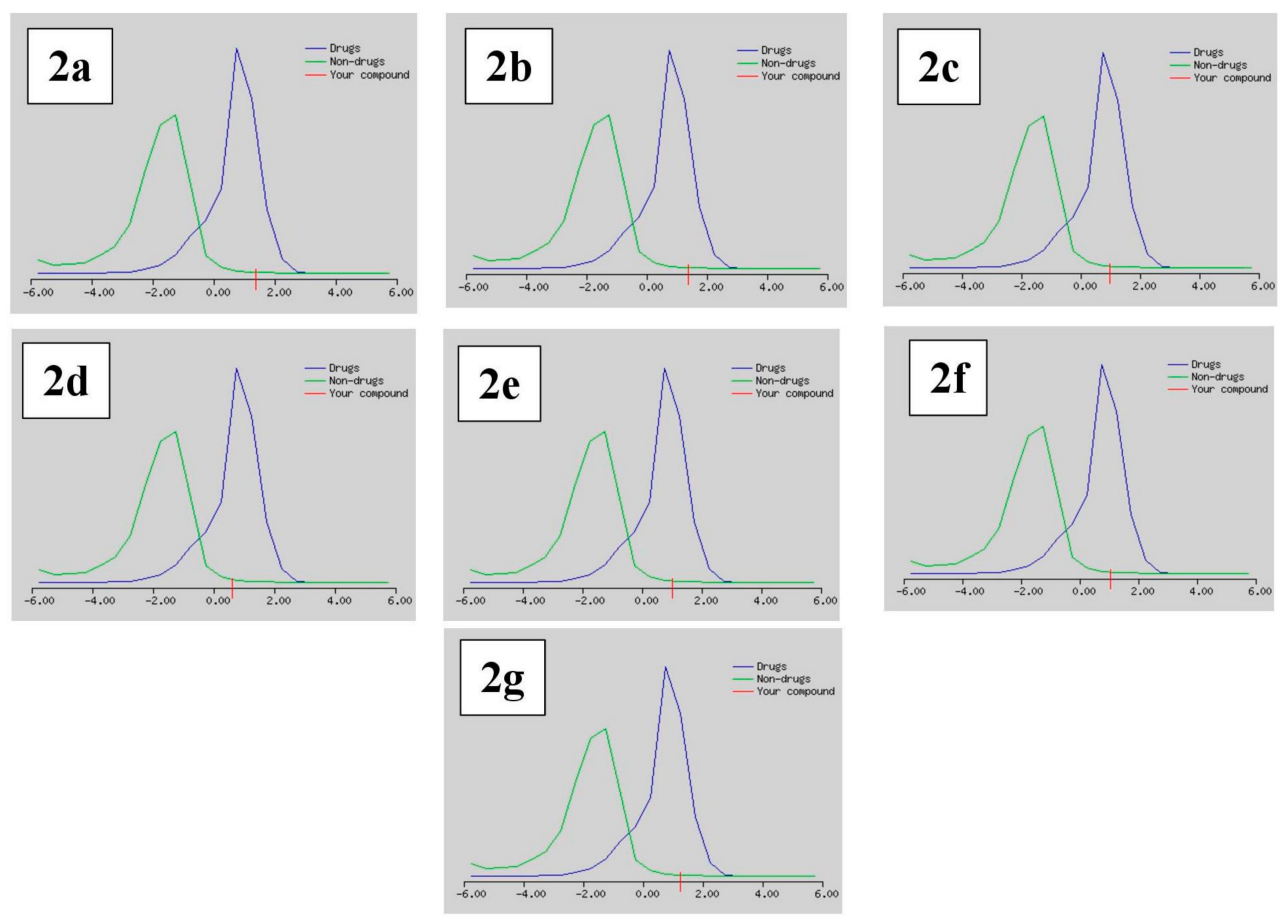
Drug likeness score model of the selected compounds **2a**–**2g**.

Drug likeness was deemed an imperative attribute that serves as a basis in order to establish whether the derivatives could be influential drug nominees. The Lipinski [47], Ghose [48], Veber [49], Egan [50], and Muegge [51] rules were implemented to appraise drug likeness and foretell whether a drug candidate was likely to be bioactive according to several vital criterion like molecular weight, LogP, and the number of hydrogen bond acceptors (HBA) as well as the number of hydrogen bond donors (HBD). The number of violations to the above-mentioned rules along with their bioavailability and drug likeness score are documented in Table 4. The Lipinski (Pfizer) sieve was the trendsetter rule-of-five (RO5), except for compounds **2c** and **2g**, which had one and two of Lipinski’s violations, respectively; the rest of all the tested compounds were drug-like. The forecasting process with the Ghose rule flaunted that Compound **2c** was discarded with one violation, compounds **2d** and **2g** were declined with two violations, and the rest of all the compounds upheld the rule. According to the appraisal procedure with the Veber and Egan rules, all compounds were alike except for compounds **2e** and **2g**, with one violation each. Moreover, the scrutinizing approach in accordance to the Muegge rules displayed that all the compounds satisfied the benchmark of the drug likeness assessment except for compounds **2d**, **2e**, and **2g**, each with one violation. Meanwhile, these novel compounds exhibited a bioavailability score of 0.55, except for **2g**, whose bioavailability score was 0.11. Furthermore, all the screened candidates exhibited good drug-likeness scores, ranging from 0.62 to 1.37, as presented in Figure 5.

### 2.3. Biological Screening

#### 2.3.1. Antimicrobial Screening

**Figure 6:**
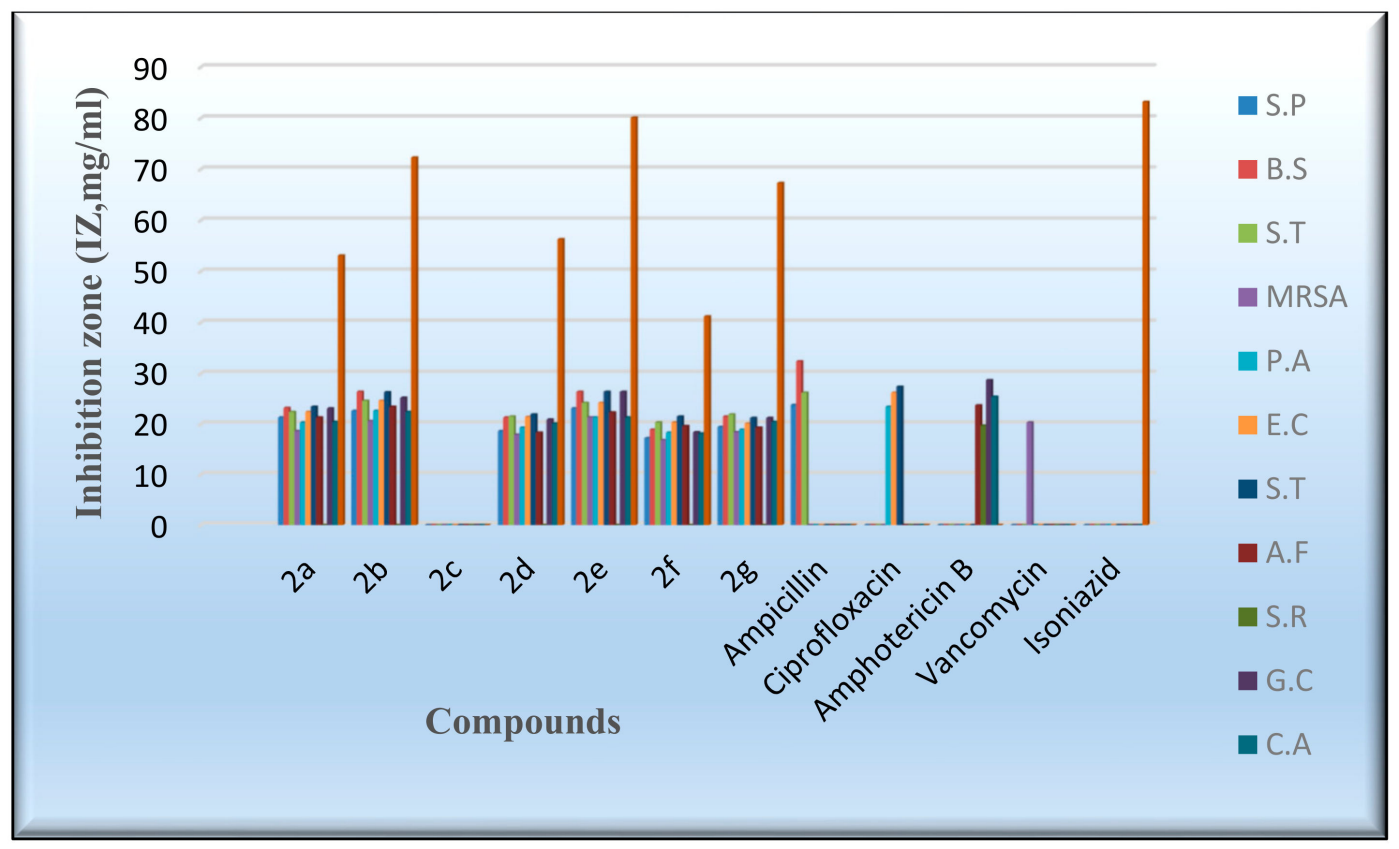
Evaluation of the IZ values of the synthesized derivatives.

**Figure 7:**
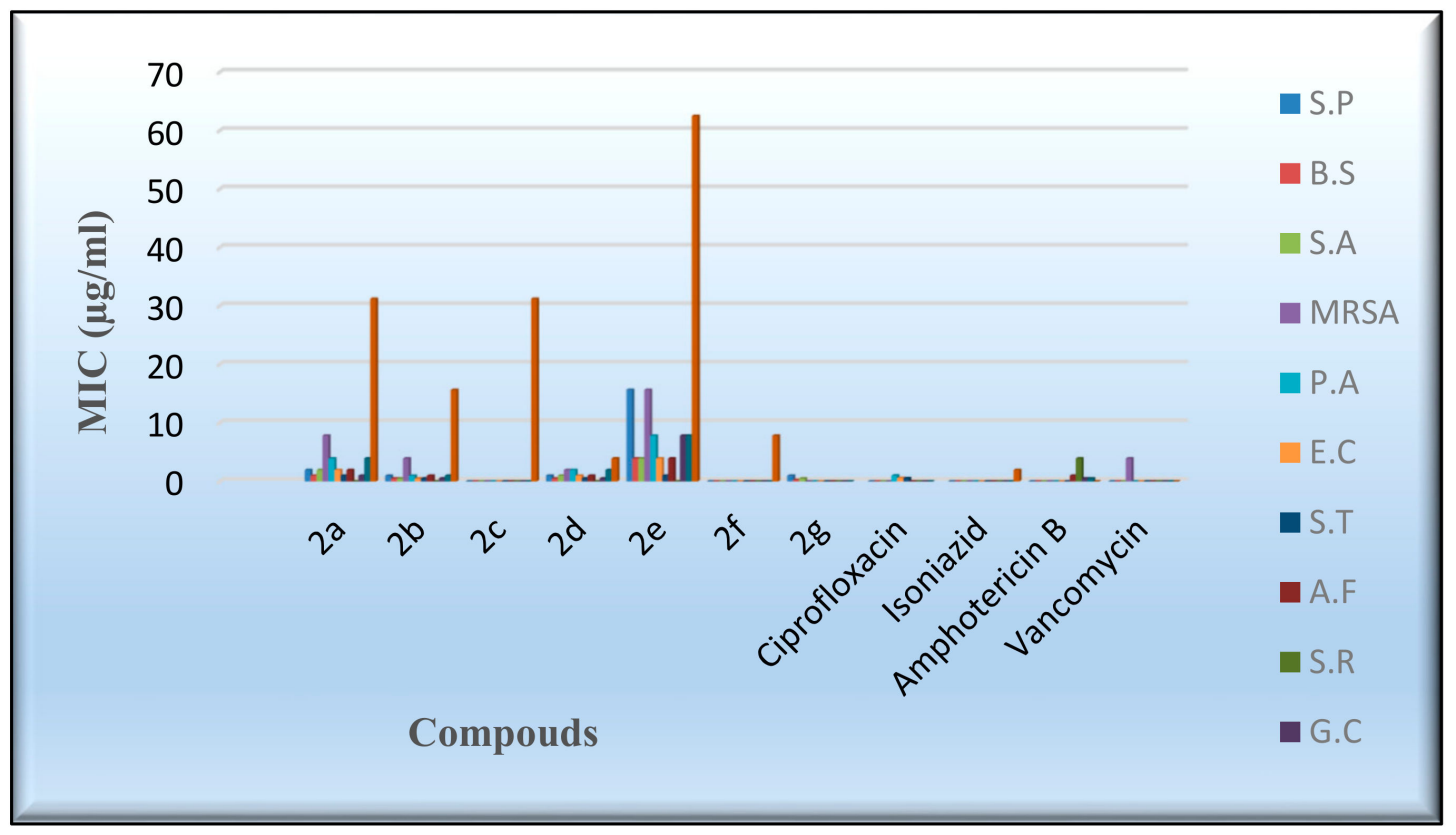
Evaluation of the MIC values of the desired compounds.

An in vitro antimicrobial assay was performed on the targeted molecules **2a**–**2g** in order to evaluate their antibacterial, antifungal, and antimycobacterium characters through the agar diffusion-well method [52]. The activities of the novel derivatives were investigated against seven human pathogens, including four Gram-positive bacteria, three Gram-negative bacteria, four fungi, and *Mycobacterium tuberculosis*, Table 5 and Table 6. ampicillin, gentamicin, amphotericin B, vancomycin, and isoniazid were exploited as control drugs [53,54]. The employment of the serial dilution procedure identified the inhibition zones and the minimum inhibitory concentrations (MIC), as shown in Figure 6 and Figure 7. The values of the inhibition zone (IZ) and the minimum inhibitory concentrations (MIC) of the target compounds against *Pseudomonas aeruginosa* and *E. coli* point out a high inhibitory activity associated with derivatives **2a**,**2b** and **2d**–**2g**, as indicated by IZ values ranging from 18.3 to 24.6 mm and the MIC values from 0.49 to 3.9 µg/mL, as shown in Table 5 and Table 6. Meanwhile, the results of the inhibition zone (IZ) and the minimum inhibitory concentrations (MIC) were discovered to be more effective against *Salmonella typhimurium* by the IZ value of 23.4–26.4 mm. Figure 6 and Figure 7 display the aforementioned data. The antibacterial activity of most compounds was found to be comparably active to the standard drugs when evaluated against the examined gram-positive bacteria, with a MIC range of 0.49 to 3.9 µg/mL. Compounds **2b** and **2e** exhibited a mild inhibitory activity against the Methicillin-resistant *Staphylococcus aureus* (MRSA), as shown by the IZ value of 21.3–20.6 mm and the MIC value of 1.95–3.9 µg/mL. Meanwhile, most of the investigated derivatives were slightly more active against the *Tuberculosis* (TB) and the fungal species. Compound **2e** demonstrated moderate activity in comparison to isoniazid. On the other hand, Compound **2c** did not indicate any antimicrobial activity against the tested bacteria, fungi, and TB. In general, the antimicrobial activity of the new flavanone-containing chromene derivatives presented more potency than the reference drugs against Gram-negative bacteria and a relatively reduced activity towards Gram-positive bacteria, fungi, and TB.

#### 2.3.2. Cytotoxic Screening

**Figure 8:**
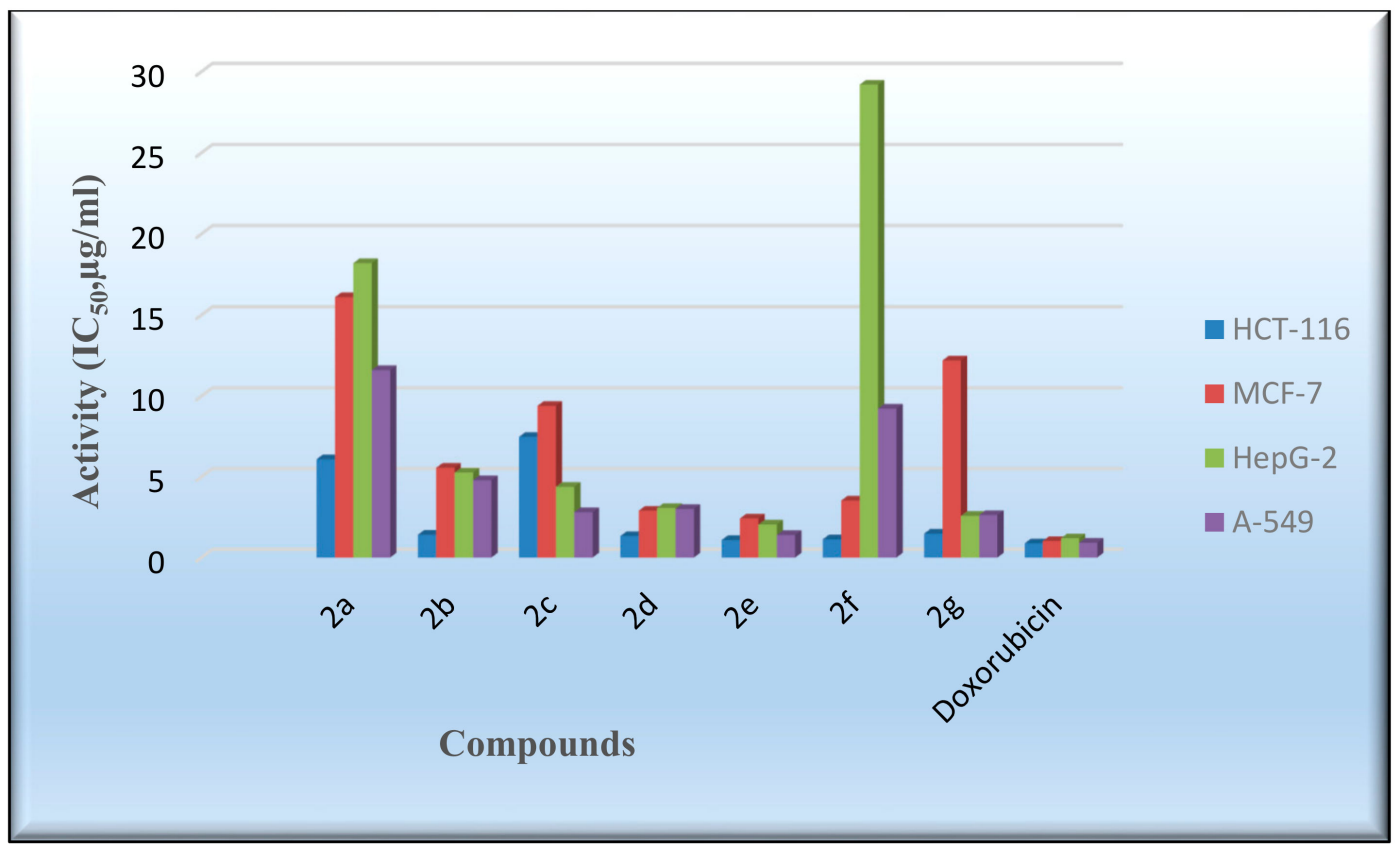
Evaluation of the cytotoxic activity of the flavanone-containing chromenes. HCT-116; MCF-7; HepG-2; A-549.

The in vitro cytotoxicity of the produced derivatives **2a**–**2g** was tested with the 2-(4,5-dimethylthiazol-2-yl)-3,5-diphenyl-2Htetrazol-3-ium bromide (MTT) method against the human colon carcinoma (HCT-116), human hepatocellular carcinoma (HepG-2), human breast adenocarcinoma (MCF-7), and adenocarcinoma human alveolar basal epithelial cell (A-549) cell lines [55,56]. The standard reference drug utilized was doxorubicin, and the inhibitory activities (IC_50_) are provided in Table 7 and Figure 8. The compounds **2b** and **2d**–**2g** showed good cell growth inhibitory behavior against the HCT-116 cell line (IC_50_ = 1.08–1.48 µg/mL). The IC_50_ values of the assessed compounds against the HepG-2 and A-549 cell lines indicated that Compound **2e** had a comparable activity with respect to the reference drug. By evaluating the inhibitory activity of the targeted molecules with that of doxorubicin against the MCF-7 cell line, it was determined that most of the compounds were less active than doxorubicin. Further research might lead to enhanced inhibitory behavior, especially for Compound **2e**, against the HCT-116 cell line.

The cell viability of colon, breast, liver, and epithelial cell lines, following exposure to different concentrations of compounds **2a**–**2g**, was assessed with the MTT method. Data are presented as IC_50_ (µg/mL) values.

### 2.4. Molecular Docking and SAR Analysis

#### 2.4.1. Docking Studies

**Figure 9:**
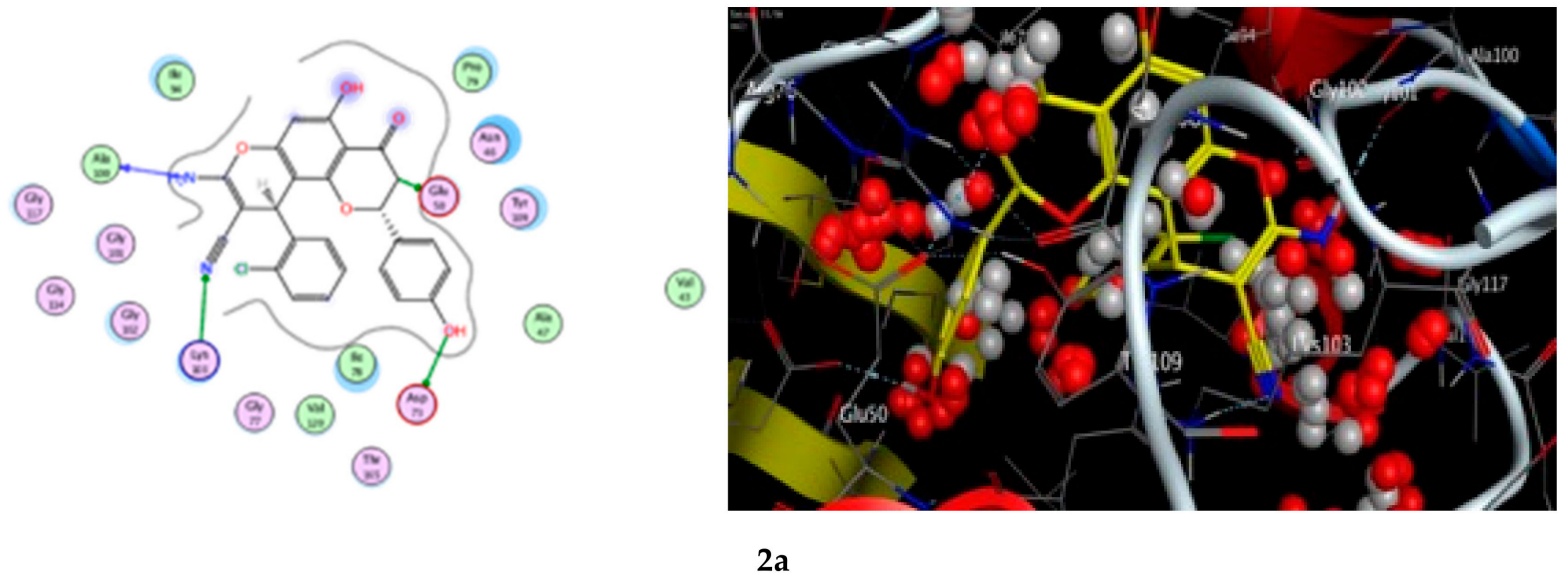

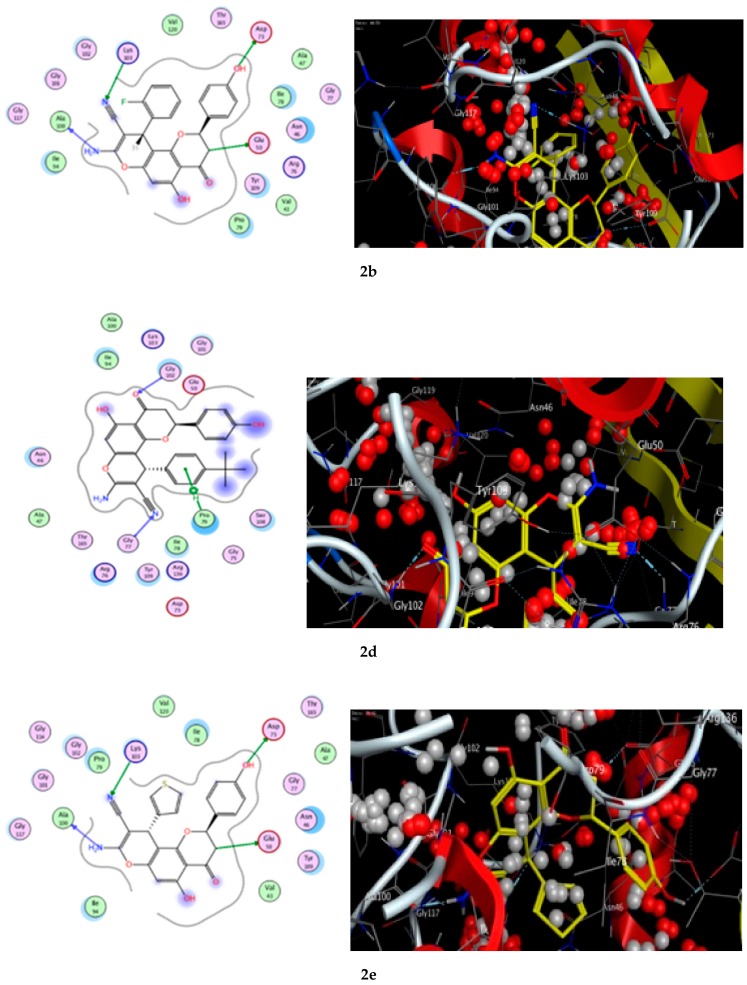
The 2D and 3D binding interaction of the target compounds; (**2a**,**2b**,**2d**, and **2e**) inside the DNA gyrase enzyme subunit B. (**2a**,**2b**,**2d**,**2e**). (The compound structures in this figure are 3D molecular structures where blue color is associated with nitrogen, yellow with carbon, red with oxygen, white with hydrogen and green with halogen atoms).

A docking study was performed for the tested compounds **2a**, **2b**, **2d**, and **2e** in order to scrutinize their possible interactions with gyrase B (GyrB43) from *E. coli* (PDB code: 4PRV; resolution 2.00 Å) by employing the docking module that was implemented in the MOE software. The bacterial DNA gyrase has been reported as a good target for antibacterial agents and the assessment of potential antibacterial activity of new compounds [18]. Figure 9 presents the best docking poses for the novel flavanone bearing chromene moiety inside the GyrB binding pocket.

The results of the docking experiments are portrayed in Table 8. The binding map of the derivatives in the pocket is explicated through different fragments: 4-C=O, 5-OH, 4′-OH, 8-NH_2_, 3-CH, 9-CN, and phenyl. These fragments formed stable hydrogen bonding interactions with a panel of corresponding pocket residues: Ala 100, Glu 50, Asp 73, Lys 103, Gly 77, Gly 102, and Pro 79.

**Figure 10:**
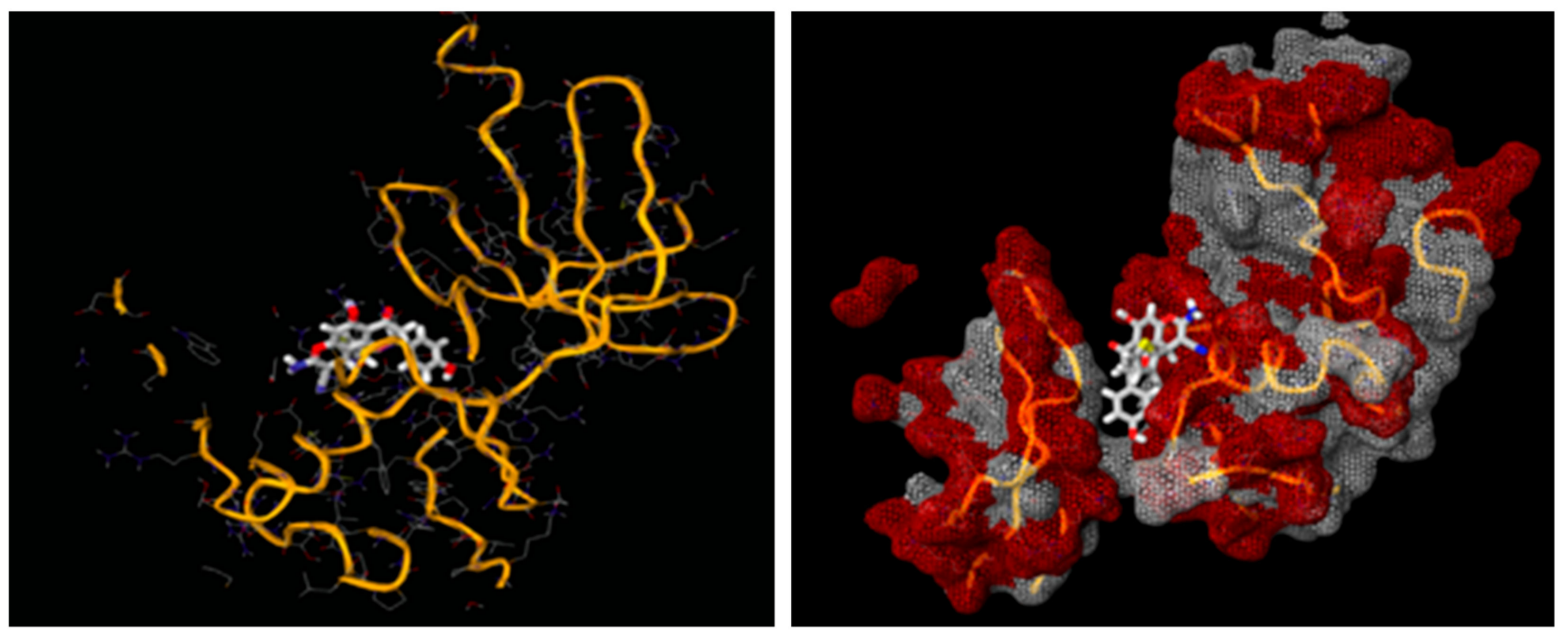
Docking of Compound **2e** into the active site of the cyclin-dependent kinase-2 (CDK2, PDB ID: 1FVV). (The compound **2e** structure in this figure is a 3D molecular structure where blue color is associated with nitrogen, grey with carbon, red with oxygen, yellow with sulfur, white with hydrogen atoms).

In order to verify the supreme in silico conformation, the molecular docking screenings of the novel derivatives were rendered with the cyclin-dependent kinase-2 (CDK2, PDB ID: 1FVV) as the target protein. To entertain this intent, a Lamarckian genetic algorithm docking program, AutoDock 4.0, was engaged where the flavanone chromene derivatives were docked on the target receptor. The docking scores of all ligands are documented in Table 9. The ligands unveiled low-to-moderate docking scores towards the target receptor CDK2 that ranged from −7.0 to −5.5. The docking pattern of the selected ligand derivative **2e**, which exhibited the highest docking score, is illustrated in Figure 10.

#### 2.4.2. Molecular Descriptors-Based SAR Analysis

**Figure 11:**
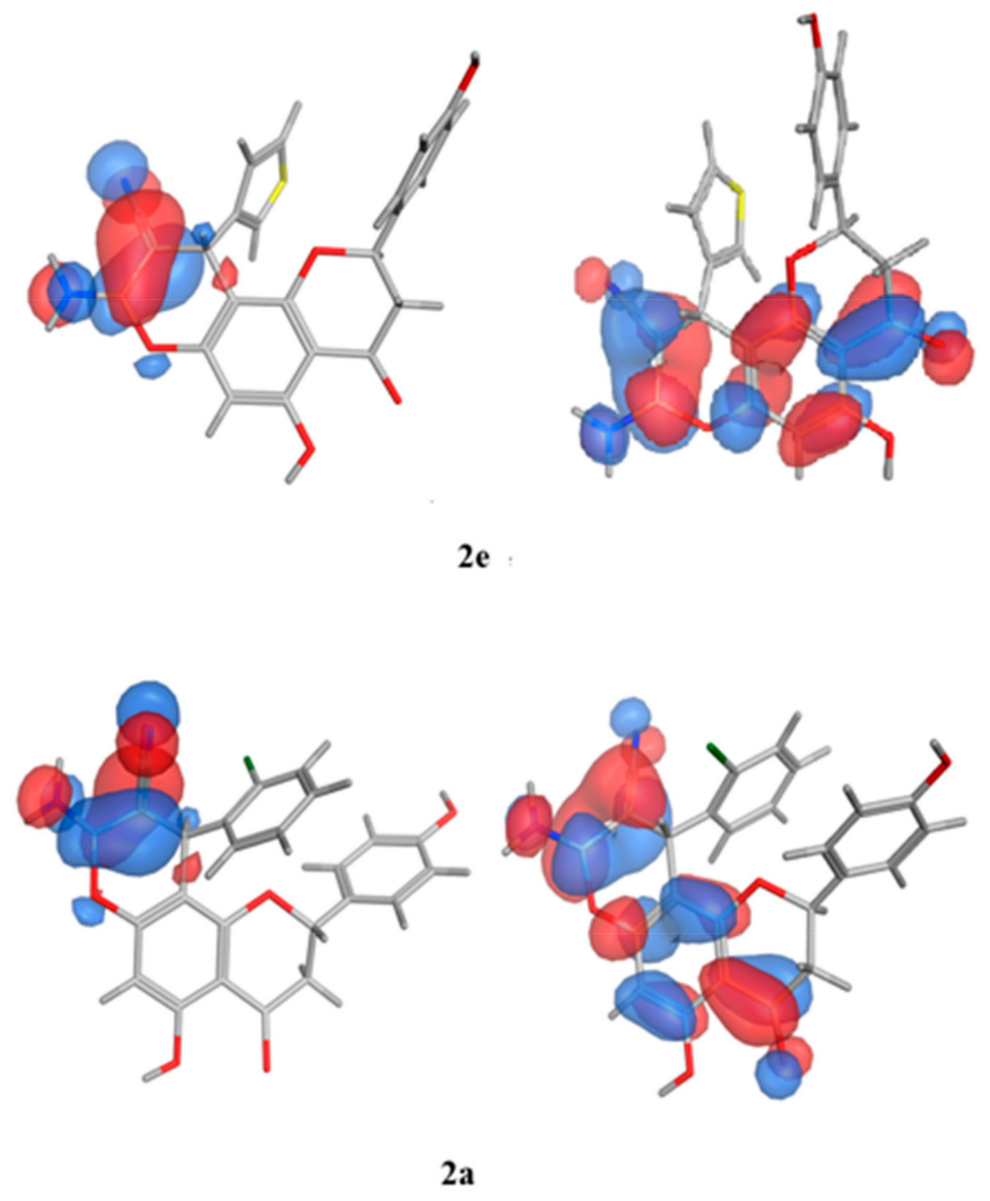

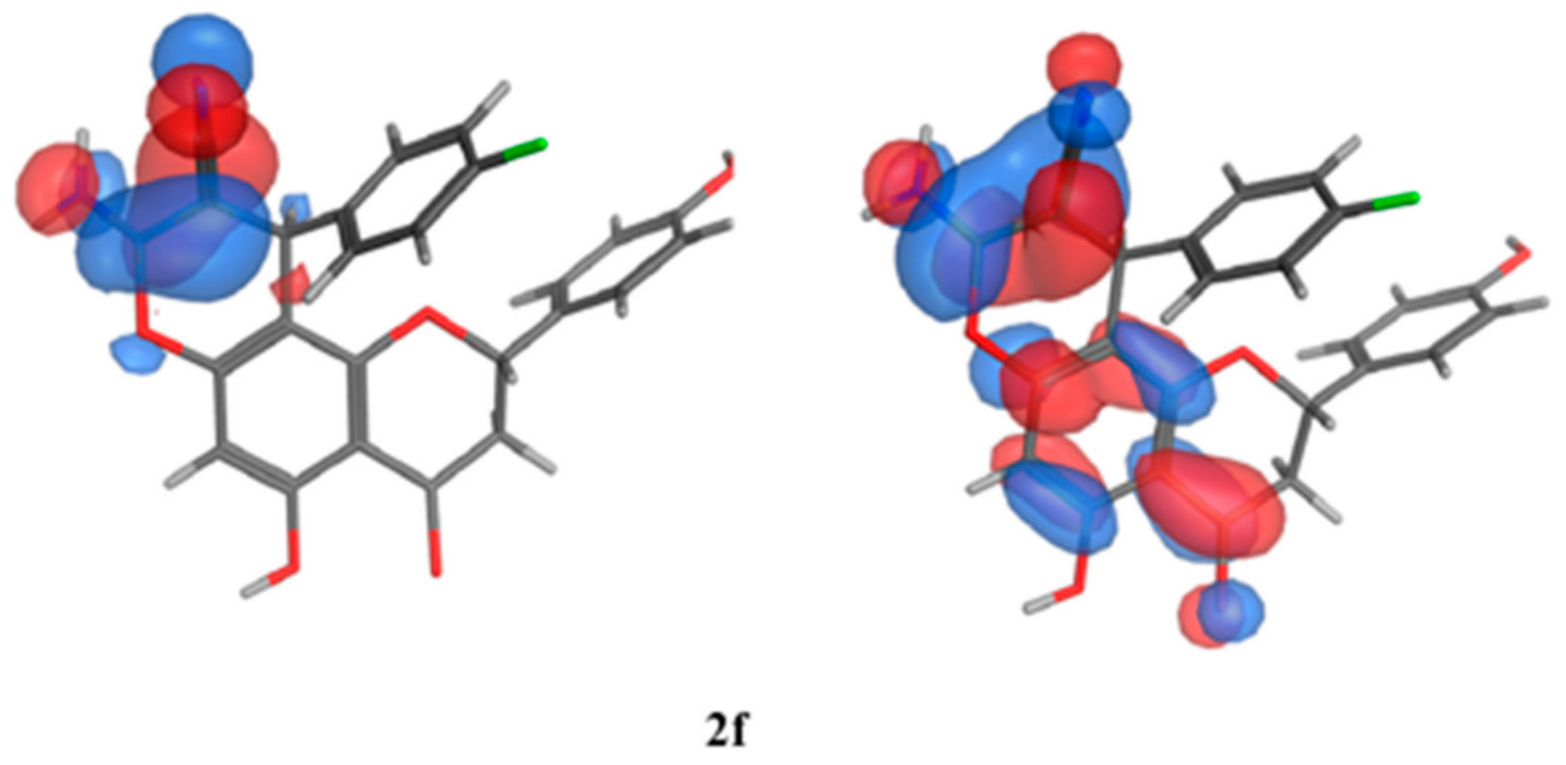
Molecular orbital spatial distribution and localization for the HOMO and LUMO of three representative compounds (**2e**,**2a**,**2f**). (The compound structures in this figure are 3D molecular structures where blue color is associated with nitrogen, grey with carbon, red with oxygen, yellow with sulfur, white with hydrogen and green with halogen atoms).

The SAR analysis was implemented on a collection of molecules (**2e**, **2d**, and **2a**) that exhibited diverse activity profiles via the quantum mechanical calculations, and the analysis employed the Molecular Orbital PACkage (MOPAC) quantum engine in the MOE software [57]. For numerous pharmacological and chemical procedures, their significance was held within the energies of their frontier orbitals, which delivered data on the electron-donating and electron-withdrawing nature of derivatives with the formation of a charge transfer complex (CTC) [58], as illustrated in Table 10. Furthermore, the institution of the electron-accepting centers to the pursued moiety showed an encouraging impact against the antimicrobial behavior, specifically towards the Gram-negative bacteria, as established by the quantum estimates. As can be seen in Figure 11, the representative molecules demonstrating dissimilarities in the activity profiles was due to the electron density allocation of the Highest Occupied Molecular Orbital (HOMO) and the lowest Unoccupied Molecular Orbital (LUMO) surfaces. Regarding the newly active molecules, the substituent distribution resulted in a lower density quality in their HOMO in comparison to their LUMO analogue. Moreover, it was discovered that the compounds experiencing lower levels of activity possessed efficacious electron donors that interacted with several targets through the charge transfer mechanism prior to the attainment of the enzyme, and they could not pass through the bacterial cell membranes.

**Figure 12:**
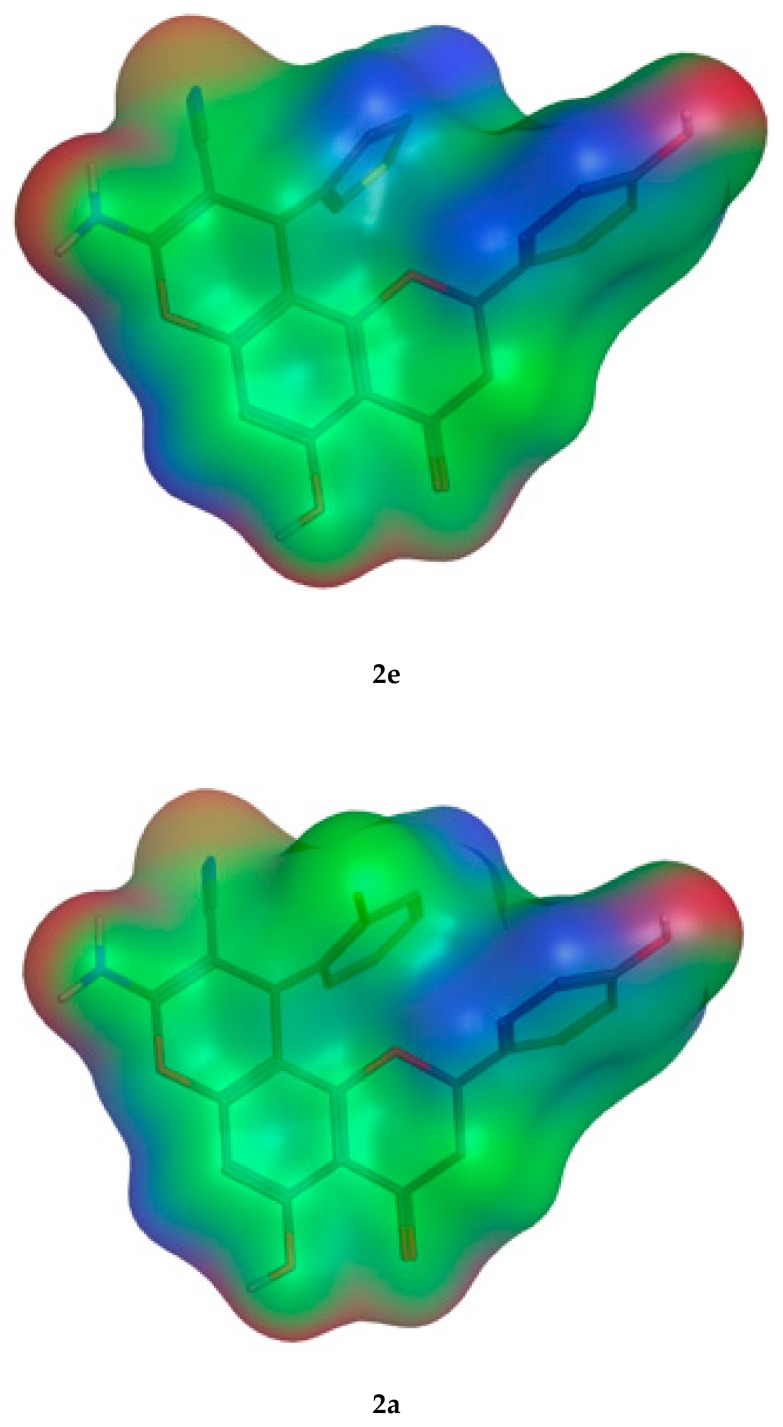

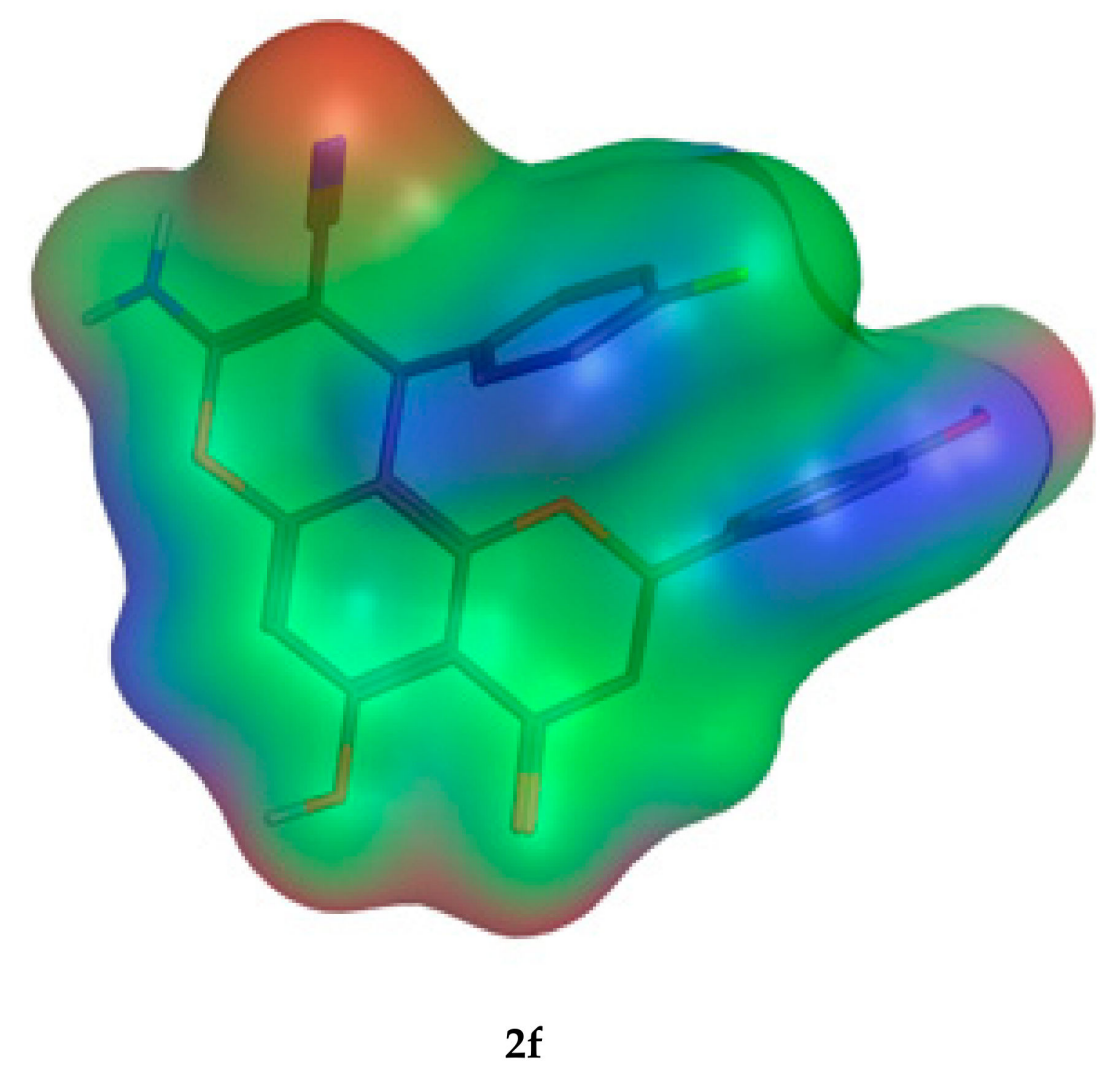
The 2D molecular surface differences for the three representative compounds (**2e**,**2a**,**2f**). The green color indicates the hydrophobic regions, and the red and blue denote the hydrophilic polar areas.

The molecular parameters, including the number of atoms, orbitals, electrons, Self Consistent Field (SCF) energy, the dipole moment, and the heat of formation, were calculated from the Molecular Operating Environment (MOE) program. These values were reported for the representative derivatives of high activity (**2e**), medium activity (**2a**), and low activity (**2f**). Moreover, the electrostatic maps were analyzed against the molecular surfaces for the novel molecules (Figure 12), where the green regions show the hydrophobic moieties and the red/blue regions show the polar ones.

## 3. Materials and Methods

### 3.1. Method of Computation: In Silico Study

The bioactivity scores of the synthesized compounds, **2a**–**2g**, were evaluated by utilizing the Molinspiration Cheminformatics server (http://www.molinspiration.com). The Molsoft server (http://www.molsoft.com) was employed to find the drug likeness scores of the above-mentioned compounds. The molecular descriptors/physico-chemical and pharmaco-kinetic/ADME attributes of the synthesized compounds, **2a**–**2g**, were determined with the SwissADME server (http://www.sib.swiss). The same web interface was used to carry out the investigations of the drug likeness violations of compounds **2a**–**2g**.

### 3.2. Materials and Instrumentation

The chemicals and solvents were purchased from Sigma-Aldrich (Oakville, ON, Canada) and Alfa Aesar (Tewksbury, MA, USA), and they were used as received. Compound **1** was purchased from Sigma-Aldrich (Canada). The melting points were determined in open capillaries by using a Stuart Scientific electrothermal apparatus (Stuart Scientific, Stone, SFD, UK), and they were uncorrected. The progress of the reactions was monitored by employing thin layer chromatography (TLC) on Merck silica gel 60 F254 plates. The infrared (IR) spectra were recorded by utilizing a Bruker Alpha FT-IR Spectrometer (Bruker, Billerica, MA, USA) as pressed KBr pellets. The ^1^H-NMR and ^13^C-NMR spectra were recorded at 300 and 75 MHz, respectively, on a Bruker Avance Spectrometer (Bruker, Billerica, MA, USA) in DMSO-*d*_6_ with tetramethylsilane as an internal standard. The elemental analyses for C, H, and N were performed by using an Exeter Analytical, Inc. CE-440 Elemental Analyzer (Chelmsford, MA, USA) [59].

### 3.3. Synthesis

The general procedure for the synthesis of 8-amino-10-phenyl-5-hydroxy-2-(4-hydroxy-phenyl)-4-oxo-3,4-dihydro-2*H*,10*H*-pyrano[2,3-f]chromene-9-carbonitrile derivatives **2a**–**2g** is as follows. 

Aryl aldehyde (2.3 mmol) was added to a stirred solution that contained Compound **1** (2.3 mmol) in 5 ml of ethanol and malononitrile or ethyl cyanoacetate (2.3 mmol) with a few drops of piperidine. The reaction mixture was stirred at reflux. After the completion of the reaction (monitored by TLC), the mixture was kept at room temperature, and the formed solid product was collected by filtration and washed with ethanol and hexane to yield **2a**–**2g**.

*8-amino-10-(2-chlorophenyl)-5-hydroxy-2-(4-hydroxy-phenyl)-4-oxo-3,4-dihydro-2H,10H-pyrano[2,3-f] chromene-9-carbonitrile* (**2a**). White solid (0.23 g, 27.45%), m.p. 262 °C; IR (KBr) cm^−1^: 3489 (OH), 3360 (NH_2_), 2178 (CN), and 1634 (C=O); ^1^H-NMR (DMSO, 300 MHz) δ 12.3 (s, 1H, C_5_–OH), 9.63 (s, 1H, C_4_′–OH), 7.39 (d, *J* = 7.5 Hz, 1H, C_2_″–H), 7.33 (d, *J* = 8.5 Hz, 2H, diastereomeric C_2_′–H), 7.32 (d, *J* = 8.5 Hz, 2H, diastereomeric C_2_′–H), 7.29–7.18 (m, 2H, C_4_″–H and C_4_″–H), 7.15–7.04 (m, 3H, overlapping Ar–C_6_″–H and NH_2_), 6.80 (d, *J* = 8.5 Hz, 2H, C_3_′–H), 6.23 (s, 1H, C_6_–H), 5.55 (dd, *J* = 12.8, 2.8 Hz, 1H, diastereomeric C_2_–H), 5.53 (dd, *J* = 12.8, 2.8 Hz, 1H, diastereomeric C_2_–H), 5.08 (s, 1H, C_10_–H), 3.40 (dd, *J* = 17.1, 13.0 Hz, 1H, diastereomeric *trans* C_3_–H), 3.41 (dd, *J* = 17.1, 13.0 Hz, 1H, diastereomeric *trans* C_3_–H), 2.73 (dd, *J* = 17.1, 2.7 Hz, 1H, diastereomeric *cis* C_3_–H), and 2.70 (dd, *J* = 17.1, 2.7 Hz, 1H, diastereomeric *cis* C_3_–H); ^13^C-NMR (DMSO, 75 MHz) δ 198.9 (C=O), 162.1 (C_5_–O), 160.5 (C_13_–O), 160.2 (C_8_–O), 158.8 (C_4_′–O), 156.6 (C_12_–O), 142.6 (diastereomeric C_1_″), 142.5 (diastereomeric C_1_″), 132.9 (C–Cl), 132.8 (C_1_′), 131.3 (diastereomeric CH), 131.2 (diastereomeric CH), 130.4 (C_3_″–H), 129.4 (C_2_′–H), 129.2 (CH), 128.4 (CH), 120.3 (CN), 116.1 (C_3_′–H), 105.9 (C_14_), 103.8 (C_11_), 95.7 (diastereomeric C_6_–H), 95.6 (diastereomeric C_6_–H), 79.8 (diastereomeric C_2_–H), 79.7 (diastereomeric C_2_–H), 56.7 (diastereomeric C_9_), 56.6 (diastereomeric C_9_), 43.0 (diastereomeric C_3_), 42.7 (diastereomeric C_3_), and 34.0 (C_10_–H); Anal. Calcd for C_25_H_17_N_2_O_5_Cl: C, 65.15; H, 3.72; N, 6.08. Found: C, 65.34; H, 3.63; N, 6.02.

*8-amino-10-(2-fluorophenyl)-5-hydroxy-2-(4-hydroxy-phenyl)-4-oxo-3,4-dihydro-2H,10H-pyrano[2,3-f] chromene-9-carbonitrile* (**2b**). Yellow solid (0.42 g, 57.76% ), m.p. 290 °C; IR (KBr) cm^−1^: 3497 (OH), 3403, 3192 (NH_2_), and 2198 (CN), 1647 (C=O); ^1^H-NMR (DMSO, 300 MHz) δ 12.4 (s, 1H, C_5_–OH), 9.63 (s, 1H, C_4_′–OH), 7.32 (d, *J* = 8.5 Hz, 1H, diastereomeric C_2_′–H), 7.31 (d, *J* = 8.5 Hz, 2H, diastereomeric C_2_′–H), 7.29–7.20 (m, 1H, C_4_″–H), 7.20–7.11 (m, 3H, C_3_″–H, C_5_″–H and C_6_″–H), 7.09 (s, NH_2_), 6.80 (d, *J* = 8.5 Hz, 2H, C_3_′–H), 6.22 (s, 1H, C_6_–H), 5.55 (dd, *J* = 12.2, 2.7 Hz, 1H, diastereomeric C_2_–H), 5.52 (dd, *J* = 12.6, 2.5 Hz, 1H, diastereomeric C_2_–H), 4.82 (s, 1H, C_10_–H), 3.51–3.37 (m, 1H, diastereomeric *trans* C_3_–H), 2.73 (dd, *J* = 17.1, 2.7 Hz, 1H, diastereomeric *cis* C_3_–H), and 2.70 (dd, *J* = 17.1, 2.7 Hz, 1H, diastereomeric *cis* C_3_–H); ^13^C-NMR (DMSO, 75 MHz) δ 198.0 (C=O), 162.6 (dd, *J* = 230 Hz, C–F), 162.2 (C_5_–O), 159.5 (C_13_–O), 159.3 (C_8_–O), 157.9 (C_4_′–O), 155.7 (C_12_–O), 129.9 (C_1_″), 128.5 (C_2_′–H), 128.3 (C_1_′), 128.2 (C_4_″–H), 124.5 (C_5_″–H), 119.7 (CN), 115.5 (d, *J* = 21.0 Hz, diastereomeric C_3_″), 115.2 (C_3_′–H), 115.1 (d, *J* = 21.0 Hz, diastereomeric C_3_″), 107.9 (diastereomeric C_14_), 104.7 (diastereomeric C_14_), 102.8 (diastereomeric C_11_), 100.1 (diastereomeric C_11_), 95.8 (minor atropic isomer C_6_–H), 95.6 (minor atropic isomer C_6_–H), 94.8 (diastereomeric C_6_–H), 94.8 (diastereomeric C_6_–H), 79.0 (diastereomeric C_2_–H), 78.8 (diastereomeric C_2_–H), 78.6 (minor atropic isomer C_2_–H), 56.0 (diastereomeric C_9_), 55.7 (diastereomeric C_9_), 42.2 (diastereomeric C_3_), 41.8 (diastereomeric C_3_), 30.3 (diastereomeric C_10_), and 30.3 (diastereomeric C_10_); Anal. Calcd for C_25_H_17_N_2_O_5_F: C, 67.57; H, 3.86; N, 6.30. Found: C, 66.85; H, 3.98; N, 6.09.

*8-amino-10-(3-bromophenyl)-5-hydroxy-2-(4-hydroxy-phenyl)-4-oxo-3,4-dihydro-2H,10H-pyrano[2,3-f] chromene-9-carbonitrile* (**2c**). White solid (0.46 g, 50.07%), m.p. 182 °C; IR (KBr) cm^−1^: 3634(OH), 3424 (NH_2_), 2207 (CN), and 1653 (C=O); ^1^H-NMR (DMSO, 300 MHz) δ 12.4 (s, 1H, C5–OH), 9.63 (s, 1H, C4′–OH), 7.45–7.37 (m, 2H, H2′ and H6′), 7.36–7.23 (m, 3H, H2″, H4″ and H5″), 7.21–7.10 (m, 3H, H6″ and NH_2_), 6.80 (d, *J* = 8.5 Hz, 2H, H3′ and H5′), 6.24 (s, 1H, H6), 5.56 (dd, *J* = 12.5, 2.8 Hz, 1H, diastereomeric H2), 5.53 (dd, *J* = 13.1, 2.8 Hz, 1H, diastereomeric H2), 4.62 (s, 1H, diastereomeric H10), 4.61 (s, 1H, diastereomeric H10), 3.42 (dd, *J* = 17.2, 13.5 Hz, 1H, diastereomeric *trans* H3ax), 3.41 (dd, *J* = 17.6, 13.3 Hz, 1H, diastereomeric *trans* H3ax), 2.75 (dd, *J* = 17.4, 3.0 Hz, 1H, diastereomeric *cis* H3eq), and 2.71 (dd, *J* = 17.1, 2.7 Hz, 1H, diastereomeric *cis* H3eq); ^13^C-NMR (DMSO, 75 MHz) δ 198.9 (C=O), 162.9 (C-5), 160.4 (C-13), 160.3 (C-8), 158.8 (C-4′), 156.2 (C-12), 148.5 (C-1′’), 131.7 (C-2′’), 130.6 (C-5′’), 129.4 (C-2′ and C-6′), 129.2 (C-1′), 128.5 (C-4′’), 127.2 (C-6′’), 122.5 (C-3′’), 120.7 (CN), 116.1 (C-3′ and C-5′), 105.7 (C-14), 104.3 (C-11), 96.0 (C6–H), 79.8 (C-2), 57.7 (C-9), 43.0 (diastereomeric C-3), and 42.9 (diastereomeric C-3), 36.2 (C-10); Anal. Calcd for C_25_H_17_N_2_O_5_Br: C, 59.42; H, 3.39; N, 5.54. Found: C, 59.82; H, 3.28; N, 5.31.

*8-amino-10-(4-tert-butylphenyl)-5-hydroxy-2-(4-hydroxy-phenyl)-4-oxo-3,4-dihyd-ro-2H,10H-pyrano[2,3-f] chromene-9-carbonitrile* (**2d**). Yellowish white solid (0.20 g, 22.80%), m.p. 264 °C; IR (KBr) cm^−1^: 3488 (OH), 3381 (NH_2_), 2961– 2869 (CH), 2185 (CN), and 1643 (C=O); Diastereomeric ratio: 1:1; atropic isomeric ratio 1:4.3 due to locked conformation and hindered rotation. Major atropic isomer: ^1^H-NMR (DMSO, 300 MHz) δ 11.9 (s, 1H, C_5_–OH), 9.62 (s, 1H, C_4_′–OH), 7.30 (d, *J* = 8.3 Hz, 2H, C_3_″–H), 7.03 (br s, 2H, NH_2_), 6.94 (d, *J* = 8.2 Hz, 2H, C_2_″–H), 6.91 (d, *J* = 8.0 Hz, 2H, C_2_′–H), 6.74 (d, *J* = 8.1 Hz, 2H, C_3_′–H), 6.21 (s, 1H, C_6_–H), 5.07 (dd, *J* = 12.8, 2.5 Hz, 1H, C_2_–H), 4.46 (s, 1H, C_10_–H), 3.34 (dd, *J* = 17.1, 13.2 Hz, 1H, trans C_3_–H), and 2.75 (dd, *J* = 17.1, 2.7 Hz, 1H, cis C_3_–H); minor atropic isomer: ^1^H–NMR δ 9.56 (s, 1H, C_4_′–OH), 6.98 (d, *J* = 8.4 Hz, 2H, C_3_′–H), 6.20 (s, 1H, C_6_–H), 5.61 (dd, *J* = 12.0, 3.1 Hz, 1H, C_2_–H), 4.49 (s, 1H, C_10_–H), 3.08 (dd, *J* = 17.3, 12.1 Hz, 1H, *trans* C_3_–H), and 2.83 (dd, *J* = 17.1, 3.3 Hz, 1H, *cis* C_3_–H); major atropic isomer: ^13^C-NMR (DMSO, 75 MHz) δ 198.7 (C=O), 161.9 (C_5_–O), 160.2 (C_13_–O), 160.0 (C_8_–O), 158.7 (C_4_′–O), 156.3 (C_12_–O), 149.7 (C_4_″), 142.9 (C_1_″), 129.4 (C_2_′–H), 127.7 (C_1_′), 127.4 (C_2_″–H), 126.0 (C_3_″–H), 120.9 (CN), 115.9 (C_3_′–H), 106.2 (C_14_), 104.4 (C_11_), 96.8 (C_6_–H), 79.9 (C_2_–H), 58.0 (C_9_), 41.7 (C_3_–H_2_), 36.2 (C_10_–H), and 35.0 (t-Bu quaternary carbon), 32.0 (CH_3_); minor atropic isomer: ^13^C-NMR δ 198.1 (C=O), 161.7 (C_5_–O), 159.7 (C_8_–O), 158.2 (C_4_′–O), 142.8 (C_1_″), 108.8 (C_14_), 104.6 (C_11_), 96.5 (C_6_–H), 79.4 (C_2_–H), 58.4 (C_9_), 42.9 (C_3_–H_2_), and 36.5 (C_10_–H); Anal. Calcd for C_29_H_26_N_2_O_5_: C, 72.18; H, 5.43; N, 5.81. Found: C, 71.99; H, 5.20; N, 5.73.

*8-amino-10-(thiophene)-5-hydroxy-2-(4-hydroxy-phenyl)-4-oxo-3,4-dihydro-2H-10H-pyrano[2,3-f] chromene-9-carbonitrile* (**2e**). White solid (0.23 g, 29.25 %), m.p. 293 °C; IR (KBr) cm^−1^: 3501 (OH), 3402, 3205 (NH_2_), 2206 (CN), and 1653 (C=O); ^1^H-NMR (DMSO, 300 MHz) δ 12.5 (s, 1H, C5–OH), 9.63 (s, 1H, C4′–OH), 7.46–7.37 (m, 1H, H4″), 7.32 (d, *J* = 8.3 Hz, 2H, H2′ and H6′), 7.20–7.14 (m, 1H, H2″), 7.11 (br s, 2H, NH_2_), 6.92–6.85 (m, 1H, H5″), 6.81 (d, *J* = 8.3 Hz, 2H, H3′ and H5′), 6.21 (s, 1H, H6), 5.59–5.43 (m, 1H, H2), 4.73 (s, 1H, diastereomeric H10), 4.71 (s, 1H, diastereomeric H10), 3.40 (dd, *J* = 17.3, 13.2 Hz, 1H*, trans* H3ax), and 2.83–2.68 (m, 1H, *cis* H3eq); ^13^C-NMR (DMSO, 75 MHz) δ 198.9 (C=O), 161.9 (C-8), 160.7 (C-13), 160.2 (C_-_5), 158.8 (C-4′), 156.3 (C-12), 146.2 (C-1″), 129.4 (C-2′ and C-6′), 129.1 (C-1′), 127.5 (diastereomeric C-5″), 127.4 (diastereomeric C-5″), 127.3 (C-4″), 121.8 (diastereomeric C-2″), 121.7 (diastereomeric C-2″), 121.0 (CN), 116.1 (C-3′ and C-5′), 106.7 (diastereomeric C-14), 106.6 (diastereomeric C-14), 105.3 (diastereomeric C-11), 105.2 (diastereomeric C-11), 96.0 (diastereomeric C-6), 95.9 (diastereomeric C-6), 79.8 (diastereomeric C-2), 79.7 (diastereomeric C-2), 57.6 (diastereomeric C-9), 57.5 (diastereomeric C-9), 43.1 (diastereomeric C-3), 42.8 (diastereomeric C-3), and 31.5 (C-10); Anal. Calcd for C_23_H_16_N_2_O_5_S: C, 63.88; H, 3.73; N, 6.48. Found: C, 63.83; H, 3.60; N, 6.16.

*8-amino-10-(4-fluorophenyl)-5-hydroxy-2-(4-hydroxy-phenyl)-4-oxo-3,4-dihydro-2H,10H-pyrano[2,3-f]chromene-9-carbonitrile* (**2f**). White solid (0.26 g, 27.31%), m.p. 230 °C; IR (KBr) cm^−1^: 3477 (OH), 3411, 3294 (NH_2_), 2914–2880 (CH), and 1686 (C=O); 1528, 1349 (NO); ^1^H-NMR (DMSO, 300 MHz) δ 12.4 (s, 1H, C5–OH), 9.63 (s, 1H, C4′–OH), 7.32 (d, *J* = 8.5 Hz, 1H, diastereomeric H2′ and H6′), 7.31 (d, *J* = 8.5 Hz, 2H, diastereomeric H2′ and H6′), 7.23–7.11 (m, 4H, H2″, H6″, H5″ and H3″), 7.10 (s, NH_2_), 6.80 (d, *J* = 8.5 Hz, 2H, H3′ and H5′), 6.23 (s, 1H, H6), 5.54 (dd, *J* = 12.8, 3.0 Hz, 1H, diastereomeric H2), 5.53 (dd, *J* = 13.1, 3.0 Hz, 1H, diastereomeric H2), 4.61 (s, 1H, diastereomeric H10), 4.59 (s, 1H, diastereomeric H10), 3.41 (dd, *J* = 17.4, 13.2 Hz, 1H, diastereomeric *trans* H3ax), 2.74 (dd, *J* = 17.1, 2.9 Hz, 1H, diastereomeric *cis* H3eq), and 2.71 (dd, *J* = 17.3, 2.9 Hz, 1H, diastereomeric cis H3eq); ^13^C-NMR (75 MHz, DMSO-d6) δ 198.82 (C=O), 163.37 (C-4″), 161.93 (C-5), 159.92 (C-13), 159.36 (C-8), 157.56 (C-4′), 154.95 (C-12), 129.91 (C-1″), 131.39.81 (C-2′ and C-6′), 130.07 (C-1′), 129.72 ( C-2″ and 6″), 128.47 (C-3″ and C-5″), 120.74 (CN), 115.99 (diastereomeric C-3′ and C-5′),116.85 (diastereomeric C-3′ and C-5′) 105.64 (C-14), 104.97 (C-11), 95.88 (diastereomeric C-6), 95.76 (diastereomeric C-6), 79.78 (diastereomeric C-2), 79.67 (diastereomeric C-2), 53.58 (C-9), and 42.75 (C-3), 36.09 (C-10); Anal. Calcd for C_25_H_17_N_2_O_5_F: C, 67.57; H, 3.86; N, 6.30. Found: C, 66.85; H, 3.98; N, 6.0.

*Ethyl-8-amino-10-(3-nitrophenyl)-5-hydroxy-2-(4-hydroxy-phenyl)-4-oxo-3,4-dih-ydro-2H,10H-pyrano[2,3-f] chromene-9-carboxlate* (**2g**). White solid (0.26 g, 27.31%), m.p. 230 °C; IR (KBr) cm^−1^: 3477 (OH), 3411, 3294 (NH_2_), 2914–2880 (CH), and 1686 (C=O); 1528, 1349 (NO); ^1^H-NMR (DMSO, 300 MHz) δ 11.9 (s, 1H, C5–OH), 9.71 (s, 1H, minor atropic isomer C4′–OH), 9.70 (s, 1H, diastereomeric C4′–OH), 9.64 (s, 1H, minor atropic isomer C4′–OH), 9.62 (s, 1H, diastereomeric C4′–OH), 8.08–7.98 (app t (two overlapped diastereomeric C_4_″–H doublets), *J* = 7.3 Hz, 1H, C_4_″–H), 7.88 (s, 1H, diastereomeric C_2_″–H), 7.79 (br s, 2H, NH_2_ and diastereomeric C_2_″–H), 7.62–7.35 (m, 2H, C_5_″–H and C_6_″–H), 7.17 (d, *J* = 9.2 Hz, 2H, minor atropic isomer C_2_′–H), 7.11 (d, *J* = 8.7 Hz, 2H, diastereomeric C_2_′–H), 7.06 (d, *J* = 8.7 Hz, 2H, diastereomeric C_2_′–H), 6.79 (d, *J* = 8.4 Hz, 2H, diastereomeric C_3_′–H), 6.72 (d, *J* = 8.6 Hz, 2H, diastereomeric C_3_′–H), 6.27 (s, 1H, diastereomeric C_6_–H), 6.26 (s, 1H, diastereomeric C_6_–H), 5.64 (dd, *J* = 12.5, 2.9 Hz, 1H, diastereomeric C_2_–H), 5.07 (dd, *J* = 13.6, 2.5 Hz, 1H, diastereomeric C_2_–H), 5.14 (dd, *J* = 13.6, 2.7 Hz, 1H, minor atropic isomer C_2_–H), 4.91 (s, 1H, minor atropic isomer C_10_–H), 4.90 (s, 1H, diastereomeric C_10_–H), 4.85 (s, 1H, minor atropic isomer C_10_–H), 4.83 (s, 1H, diastereomeric C_10_–H), 4.03–3.81 (m, 2H, OCH_2_CH_3_) 3.45 (dd, *J* = 17.2, 13.4 Hz, 1H, diastereomeric *trans* C_3_–H), 3.11 (dd, *J* = 17.4, 12.9 Hz, 1H, diastereomeric *trans* C_3_–H), 2.72 (dd, *J* = 17.6, 3.1 Hz, 1H, diastereomeric *cis* C_3_–H), and 2.66 (dd, *J* = 17.2, 2.7 Hz, 1H, diastereomeric *cis* C_3_–H); ^13^C-NMR (DMSO, 75 MHz) δ 198.7 (diastereomeric C=O), 198.3 (diastereomeric C=O), 168.7 (Minor atropic isomer ester C=O), 168.7 (Ester C=O), 162.1 (diastereomeric C_5_–O), 162.0 (diastereomeric C_5_–O),160.8 (diastereomeric C_13_–O), 160.7 (diastereomeric C_13_–O), 160.0 (diastereomeric C_8_–O), 159.9 (diastereomeric C_8_–O), 158.9 (diastereomeric C_4_′–O), 158.5 (diastereomeric C_4_′–O), 156.1 (C_12_–O), 149.6 (diastereomeric C–NO_2_), 149.6 (diastereomeric C–NO_2_), 148.3 (Minor atropic isomer C_1_″), 148.0 (C_1_″), 135.2 (diastereomeric C_6_″–H), 135.0 (diastereomeric C_6_″–H), 130.5 (diastereomeric C_5_″), 130.4 (diastereomeric C_5_″), 129.5 (diastereomeric C_2_′–H), 128.7 (Minor atropic isomer C_2_′–H), 128.5 (diastereomeric C_2_′–H), 128.4 (C_1_′), 123.4 (diastereomeric C_4_″), 123.3 (diastereomeric C_4_″), 122.9 (Minor atropic isomer C_4_″), 122.7 (Minor atropic isomer C_4_″), 122.0 (diastereomeric C_2_″), 121.9 (diastereomeric C_2_″), 116.9 (Minor atropic isomer C_3_′–H), 116.0 (diastereomeric C_3_′–H), 115.9 (diastereomeric C_3_′–H), 106.6 (diastereomeric C_14_), 106.7 (diastereomeric C_14_), 106.6 (Minor atropic isomer C_14_), 104.3 (C_11_), 96.7 (diastereomeric C_6_–H), 96.6 (diastereomeric C_6_–H), 80.2 (diastereomeric C_2_–H), 80.0 (diastereomeric C_2_–H), 76.8 (diastereomeric C_9_), 76.6 (diastereomeric C_9_), 59.8 (O**C**H_2_CH_3_), 43.4 (diastereomeric C_3_–H_2_), 43.0 (diastereomeric C_3_–H_2_), 35.3 (diastereomeric C_10_–H), 35.3 (diastereomeric C_10_–H), and 15.0 (OCH_2_CH_3_); Anal. Calcd for C_27_H_22_N_2_O_9_: C, 62.55; H, 4.28; N, 5.40. Found: C, 61.99; H, 3.79; N, 5.32.

### 3.4. Biological Studies

#### 3.4.1. Antimicrobial Screening

The antimicrobial assay has been performed according to the preceding reports [46,60].

#### 3.4.2. Cytotoxic Screening

Human colon carcinoma (HCT-116), human hepatocellular carcinoma (HEPG-2), adenocarcinomic human alveolar basal epithelial cell (A-549), and human breast adenocarcinoma (MCF-7) cell lines were obtained from the American Type Culture Collection (ATCC, Rockville, MD, USA). The cultivation of the tumor cell lines and the cytotoxic behavior was assessed by using the 2-(4,5-dimethylthiazol-2-yl)-3,5-diphenyl-2Htetrazol-3-ium bromide (MTT) colorimetric assay, as previously cited [55,61,62].

### 3.5. Molecular Modeling

The docking maneuver was assessed in order to dock the newly synthesized compounds into the crystal structure of the *E. coli* topoisomerase II DNA gyrase B (PDB code 4PRV). All the docking calculations were engineered by the MOE software [58], which was able to generate the docking input files and examine the docking outcomes. The protonation of the enzyme was carried out and energy was minimized by removing clorobiocin, all non-polar hydrogens, and crystal water molecules. In every case, the 100 docked structures were generated by implementing the genetic algorithm searches. The 3D structures of all the tested compounds were created in MOE, and the protonation of target ligands was performed. The energy minimization of the derivatives was done up to a 0.05 gradient, employing the GBVI/WSA dG force field. The obtained data were stored in a database as input files in MOE. The root-mean-square deviations (RMSD values) were calculated, and embryonic ligand binding fashion was plotted. The protein–ligand association plots were produced by utilizing MOE 2012.10. The MOE software was implemented to develop the surface molecular orbitals and to undertake the quantum mechanical calculations.

The in-silico docking tactics were executed to conduct structure-based drug designing in order to acquire the most potent inhibitor of CDK2 from the synthesized drug nominees. The Advanced Chemistry Development, Inc. ACD/Labs-Chemsketch program was exercised to construct the 3D atomic coordinates of the targeted ligands. The structure of CDK2 (PDB ID 1FVV) with crystallographic resolutions of less than 3.0 Å was procured from the Protein Data Bank (Source: www.rcsb.org/pdb/). The Dundee PRODRG2 server was employed to minimize the energy of the investigated ligands [63]. The autodock4 from “Auto-Dock Tools (ADT, 1.5.6)” is a graphical user interface program [64] that was utilized to prepare, run, and analyze the docking simulations. The docking stipulations were stationed to the software’s default values, and a standard paradigm was preserved and escorted throughout the docking studies. The outcomes were explained based on the PDF file and created by the software by designating the different ligands with respect to the predicted binding energy. The cluster gaining the lowest energy was identified as the most accurate solution. The 3D visualization of the ligand protein interaction was done by utilizing UCSF Chimera 1.11.2 and mcule, a web interface which exploits the WebGL/Javascript-based molecule viewer of GLmol.

## 4. Conclusions

The present report details the synthesis of new flavanone/chromene molecules that were nominated for their confirmed drug-likeness capabilities through in silico studies and in vitro assessments of their antimicrobial activity and cytotoxic effects. The pursued derivatives were produced by the Knoevenagal condensation of an aldehyde, followed by the intramolecular Michael addition reaction of adduct for the 4′5,7-trihydroxy-flavanone. The structures of the aforementioned compounds were established under their spectral and analytical data. The antibacterial assay revealed that several molecules exhibited high inhibitory activity against *P. aeruginosa* and *E. coli* in comparison to the reference drugs. Additionally, the cytotoxicity investigations were assessed against four different human carcinoma cell lines and demonstrated strong effects of the molecules, particularly against the HCT-116 tumor cell line. The molecular modeling outcomes demonstrated a powerful binding interaction of the newly synthesized derivatives in the active site of the GyrB with the SAR analysis.

## Figures and Tables

**Table 1:** The bioactivity of the selected compounds **2a**–**2g**.

Compound No.	GPCR Ligand	Ion Channel Modulator	Kinase Inhibitor	Nuclear Receptor Ligand	Protease Inhibitor	Enzyme Inhibitor
**2a**	−0.64	−0.82	−0.74	−0.07	−0.69	−0.31
**2b**	−0.63	−0.87	−0.76	0.00	−0.65	−0.33
**2c**	−0.68	−0.85	−0.79	−0.10	−0.67	−0.32
**2d**	−0.52	−0.71	−0.67	0.08	−0.53	−0.22
**2e**	−0.67	−0.98	−0.83	−0.18	−0.68	−0.39
**2f**	−0.59	−0.80	−0.71	0.02	−0.59	−0.27
**2g**	−0.59	−0.78	−0.82	0.04	−0.60	−0.32

**Table 2:** Physicochemical properties of the selected compounds **2a**–**2g**.

Comp. No.	Fraction Csp3 ^a^	No. of Rotatable Bonds	HBA ^b^	HBD ^c^	iLogP ^d^	Molar Refractivity	Log *S* ^e^	TPSA ^f^
**2a**	0.12	2	6	3	2.55	119.98	PS	125.8
**2b**	0.12	2	7	3	2.46	114.93	PS	125.8
**2c**	0.12	2	6	3	2.89	122.67	PS	125.8
**2d**	0.24	3	6	3	3.19	134.24	PS	125.8
**2e**	0.13	2	6	3	2.44	112.85	MS	154.04
**2f**	0.12	2	7	3	2.63	114.93	PS	125.8
**2g**	0.19	6	9	3	2.31	134.95	PS	174.13

^a^ The ratio of sp^3^ hybridized carbons over the total carbon count of the molecule; ^b^ number of hydrogen bond acceptors; ^c^ number of hydrogen bond donors; ^d^ lipophilicity; ^e^ Water solubility (SILICOS-IT [PS = poorly soluble, MS = moderately Soluble]); and ^f^ topological polar surface area (Å^2^).

**Table 3:** Pharmacokinetic/ADME (absorption, distribution, metabolism, and excretion) properties of compounds **2a**–**2g**.

Comp. No.	Pharmacokinetic/ADME Properties								
	GI abs ^a^	BBB Permeant ^b^	P-gp Substrate ^c^	CYP1A2 Inhibito ^d^	CYP2C19 Inhibitor ^e^	CYP2C9 Inhibitor ^f^	CYP2D6 Inhibitor ^g^	CYP3A4 Inhibitor ^h^	Log K_p_^i^
**2a**	Low	No	No	No	Yes	Yes	No	Yes	−5.74
**2b**	Low	No	No	No	Yes	Yes	No	Yes	−6.01
**2c**	Low	No	No	No	Yes	Yes	No	Yes	−5.97
**2d**	Low	No	No	No	Yes	Yes	No	Yes	−5.13
**2e**	Low	No	No	No	Yes	Yes	No	Yes	−6.22
**2f**	Low	No	No	No	Yes	Yes	No	Yes	−6.01
**2g**	Low	No	No	No	No	Yes	No	Yes	−6.3

^a^ Gastro Intestinal absorption; ^b^ blood–brain barrier permeant; ^c^ P-glycoprotein substrate; ^d^ CYP1A2: cytochrome P450 family 1 subfamily A member 2 (PDB:2HI4); ^e^ CYP2C19: cytochrome P450 family 2 subfamily C member 19 (PDB:4GQS); ^f^ CYP2C9: cytochrome P450 family 2 subfamily C member 9 (PDB:1OG2); ^g^ CYP2D6: cytochrome P450 family 2 subfamily D member 6 (PDB:5TFT); ^h^ CYP3A4: cytochrome P450 family 3 subfamily A member 4 (PDB:4K9T); and ^i^ skin permeation in cm/s.

**Table 4:** Drug likeness predictions of the selected compounds **2a**–**2g**.

Comp. No.	Lipinski Violations	Ghose Violations	Veber Violations	Egan Violations	Muegge Violations	Bioavailability Score	Drug Likeness Score
**2a**	0	0	0	0	0	0.55	1.37
**2b**	0	0	0	0	0	0.55	1.37
**2c**	1	1	0	0	0	0.55	0.98
**2d**	0	2	0	0	1	0.55	0.62
**2e**	0	0	1	1	1	0.55	1.02
**2f**	0	0	0	0	0	0.55	1.06
**2g**	2	2	1	1	1	0.11	1.24

**Table 5:** Antimicrobial activity of the synthetic compounds (inhibition zone (IZ) diameter (mm)) (1 mg/mL).

Compounds	Inhibition Zone Diameter (mm)											
	Gram-Positive				Gram-Negative			Fungi				TB
	S.P	B.S	S.T	MRSA	P.A	E.C	S.T	A.F	S.R	G.C	C.A	
**2a**	21.3	23.2	22.4	18.6	20.3	22.4	23.4	21.3	NA	23.1	20.4	53.1
**2b**	22.6	26.4	24.6	20.6	22.6	24.6	26.3	23.4	NA	25.2	22.4	72.3
**2c**	NA	NA	NA	NA	NA	NA	NA	NA	NA	NA	NA	NA
**2d**	18.6	21.3	21.5	17.9	19.3	21.4	21.9	18.3	NA	20.9	20.1	56.3
**2e**	23.1	26.4	24.2	21.3	21.3	24.2	26.4	22.3	NA	26.4	21.3	80.1
**2f**	17.2	18.9	20.3	16.8	18.3	20.3	21.5	19.6	NA	18.4	18.1	41.2
**2g**	19.4	21.5	21.9	18.4	18.9	20.1	21.2	19.3	NA	21.2	20.4	67.3
**Ampicillin**	23.8	32.4	26.2	−	−	−	−	−	−	−	−	−
**Gentamicin**	−	−	−	−	17.3	19.9	22.3	−	−	−	−	−
**Amphotericin B**	−	−	−	−	−	−	−	23.7	19.7	28.7	25.4	−
**Vancomycin**	−	−	−	20.3	−	−	−	−	−	−	−	−
**Isoniazid**	−	−	−	−	−	−	−	−	−	−	−	83.2

Mean zone of inhibition in mm from at least three experiments; NA: no activity; S.P.: *Streptococcus pneumoniae*; B.S.: *Bacillus subtilis*; S.A.: *Staphylococcus aureus*; MRSA: Methicillin-resistant *Staphylococcus aureus*; P.A.: *Pseudomonas aeruginosa*; E.C.: *Escherichia coli*; S.T.: *Salmonella typhimurium*; A.F.: *Aspergillus fumigatus* (RCMB 02568); G.C.: *Geotricum candidum* (RCMB 05097); S.R. *Syncephalastrum racemosum* (RCMB 05922); and C.A.: *Candida albicans* (RCMB 05036).

**Table 6:** Antimicrobial activity of the synthetic compounds (minimum inhibitory concentration (MIC), µg/mL).

Compounds	Minimal Inhibitory Concentration (MIC, µg/mL)											TBA
	Gram-Positive				Gram-Negative			Fungi				
	S.P	B.S	S.A	MRSA	P.A	E.C	S.T	A.F	S.R	G.C	C.A	
**2a**	1.95 ± 0.09	0.98 ±0.13	1.95 ± 0.31	7.81 ± 0.45	3.9 ± 0.53	1.95 ± 0.17	0.98 ± 0.15	1.95 ±0.23	NA	0.98 ± 0.24	3.9 ± 0.3	31.2
**2b**	0.98 ± 0.06	0.49 ± 0.05	0.49 ± 0.04	3.9 ± 0.15	0.98 ± 0.14	0.49 ± 0.06	0.49 ± 0.08	0.98 ± 0.18	NA	0.49± 0.13	0.98 ± 0.24	15.6
**2d**	NA	NA	NA	NA	NA	NA	NA	NA	NA	NA	NA	31.2
**2e**	0.98 ± 0.23	0.49 ± 0.15	0.98 ± 0.11	1.95 ± 0.09	1.95 ± 0.39	0.98 ± 0.24	0.49 ± 0.07	0.98 ±0.06	NA	0.49± 0.13	1.95 ± 0.18	3.9
**2f**	15.63 ± 0.91	3.9 ±0.34	3.9± 0.5	15.63 ± 1.21	7.81 ± 0.93	3.9 ± 0.35	0.98 ± 0.14	3.9± 0.5	NA	7.81± 0.73	7.81 ± 0.59	62.5
**2g**	NA	NA	NA	NA	NA	NA	NA	NA	NA	NA	NA	7.8
**Ampicillin**	0.49 ± 0.08	0.49 ± 0.12	0.49 ± 0.07	−	−	−	−	−	−	−	−	−
**Ciprofloxacin**	−	−	−	−	0.98 ±0.06	0.59 ± 0.05	0.56± 0.13	−	−	−	−	−
**Isoniazid**	−	−	−	−	−	−	−	−	−	−	−	1.95
**Amphotericin B**	−	−	−	−	−	−	−	0.98 ± 0.12	3.9 ± 0.3	0.49 ± 0.07	0.49 ± 0.13	−
**Vancomycin**	−	−	−	3.9 ± 0.42	−	−	−	−	−	−	−	−

Mean zone of inhibition in mm from at least three experiments; NA: no activity; S.P.: *Streptococcus pneumoniae*; B.S. *Bacillus subtilis*; S.A.: *Staphylococcus aureus*; MRSA: Methicillin-resistant *Staphylococcus aureus*; P.A.: *Pseudomonas aeruginosa*; E.C.: *Escherichia coli*; S.T.: *Salmonella typhimurium*; A.F.: *Aspergillus fumigatus* (RCMB 02568); G.C.: *Geotricum candidum* (RCMB 05097); S.R.: *Syncephalastrum racemosum* (RCMB 05922); and C.A: *Candida albicans* (RCMB 05036).

**Table 7:** Cytotoxicity of the chromene derivatives against the different cancer cell lines.

Compounds	R_1_	R_2_	IC_50_ (µg/mL)			
			HCT-116	MCF-7	HepG-2	A-549
***Naringenin***	-	−	31.1	54.9	11.4	55.1
**2a**	*CN*	2-ClC_6_H_4_	6.09	16.1	18.2	11.6
**2b**	*CN*	2-FC_6_H_4_	1.41	5.56	5.26	4.79
**2c**	*CN*	2-BrC_6_H_4_	7.48	9.4	4.38	2.81
**2d**	*CN*	4-C(CH_3_)_3_C_6_H_4_	1.33	2.9	3.07	3.01
**2e**	*CN*	C_4_H_4_S	1.08	2.42	2.04	1.39
**2f**	*CN*	4-FC_6_H_4_	1.13	3.53	29.2	9.23
**2g**	*COOCH_2_CH_3_*	3-NO_2_C_6_H_4_	1.48	12.2	2.58	2.63
**Doxorubicin**	*-*	−	0.88	1.02	1.19	0.91

**Table 8:** Description of the docking data of the selected target compounds, **2a**, **2b**, **2d**, and **2e** inside the active site of the gyrase B (Protein Data Bank (PDB) ID: 4PRV).

Compound No.		2a	2b	2d	2e
Amino acid (Distance Å)	Ala 100	−NH_2_ (2.1)	−NH_2_ (1.8)	−	−NH_2_ (1.7)
	Glu 50	−CH (3.2)	−CH (3.2)		CH (3.3)
	Asp 73	−OH (1.8)	−OH (1.8)	−	OH (1.8)
	Lys 103	−CN (2.2)	−CN (2.5)-	−	−CN (2.4)
	Gly 77	−	−	−C=O (1.7)	−
	Gly 102	−	−	−CN (1.79)	−
	Pro 79	−	−	Phenyl (2.7)	−
	Asn 46	−	−	−	−
Interaction type		H-bonding	H-bonding	H-bonding (Aromatic)	H-bonding
*∆*G (kcal/mol)		−14.10	−15.65	−15.35	−16.00

**Table 9:** Docking scores of compounds **2a**–**2g**.

Comp. No.	2a	2b	2c	2d	2e	2f	2g
**Docking score**	−6.5	−6.3	−6.8	−6.6	−7.0	−5.7	−5.5

**Table 10:** Molecular mechanics parameters for the selected target compounds.

Compound No.	2e	2d	2a
**Atoms**	47	61	50
**Atomic orbitals**	140	166	149
**Electrons**	154	176	164
**SCF energy**	−197.42	−195.74	−195.67
**Dipole moment**	6.03	6.35	7.45
**Heat of formation**	−47.58	−70.41	−58.06

## References

[1] Walters W.P., Murcko M.A. (2002). Prediction of ‘drug-likeness’. Adv. Drug Deliver. Rev..

[2] Lipinski C.A. (2000). Drug Like Properties and the Causes of Poor Solubility and Poor Permeability. J. Pharmacol. Toxicol. Methods.

[3] Palm K., Luthman K., Unge A.L., Strandlund G., Artursson P. (1996). Correlation of drug absorption with molecular surface properties. J. Pharm. Sci..

[4] Balakumar C., Ramesh M., Tham C.L., Khathi S.P., Kozielski F., Srinivasulu C., Hampannavar G.A., Sayyad N., Soliman M.E., Karpoormath R. (2018). Ligand- and structure-based in silico approaches to identify the potential kinesin spindle protein (KSP) inhibitors as anticancer agents. J. Biomol. Struct. Dyn..

[5] Butina D., Segall M., Frankcombe K. (2002). Predicting ADME properties in silico: Methods and models. Drug Discov. Today.

[6] Mody N., Tekade R.K., Mehra N.K., Chopdey P., Jain N.K. (2014). Dendrimer, liposomes, carbon nanotubes and PLGA nanoparticles: One platform assessment of drug delivery potential. AAPS Pharm. Sci. Tech..

[7] Ekins S., Waller C.L., Swaan P.W., Cruciani G., Wrighton S.A., Wikel J.H. (2000). Progress in predicting human ADME parameters in silico. J. Pharmacol. Toxicol. Methods.

[8] Zhang D., Luo G., Ding X., Lu C. (2012). Preclinical experimental models of drug metabolism and disposition in drug discovery and development. Acta Pharm. Sin. B.

[9] Lim S., Kaldis P. (2013). Cdks, cyclins and CKIs: Roles beyond cell cycle regulation. Development.

[10] Malumbres M., Barbacid M. (2009). Cell cycle, CDKs and cancer: A changing paradigm. Nat. Rev. Cancer.

[11] Peyressatre M., Prével C., Pellerano M., Morris M.C. (2015). Targeting Cyclin-Dependent Kinases in Human Cancers: From Small Molecules to Peptide Inhibitors. Cancers.

[12] Yamamoto H., Monden T., Miyoshi H., Izawa H., Ikeda K., Tsujie M., Ohnishi T., Sekimoto M., Tomita N., Monden M. (1998). Cdk2/cdc2 expression in colon carcinogenesis and effects of cdk2/cdc2 inhibitor in colon cancer cells. Int. J. Oncol..

[13] Lane M.E., Yu B., Rice A., Lipson K.E., Liang C., Sun L., Tang C., McMahon G., Pestell R.G., Wadler S.A. (2001). novel cdk2-selective inhibitor, SU9516, induces apoptosis in colon carcinoma cells. Cancer Res..

[14] Kitahara K., Yasui W., Kuniyasu H., Yokozaki H., Akama Y., Yunotani S., Hisatsugu T., Tahara E. (1995). Concurrent amplification of cyclin E and CDK2 genes in colorectal carcinomas. Int. J. Cancer.

[15] Tong W.-G., Chen R., Plunkett W., Siegel D., Sinha R., Harvey R.D., Badros A.Z., Popplewell L., Coutre S., Fox J.A. (2010). Phase I and pharmacologic study of SNS-032, a potent and selective Cdk2, 7, and 9 inhibitor, in patients with advanced chronic lymphocytic leukemia and multiple myeloma. J. Clin. Oncol..

[16] Weroha S.J., Lingle W.L., Hong Y., Li S.A., Li J.J. (2010). Specific overexpression of cyclin E·CDK2 in early preinvasive and primary breast tumors in female ACI rats induced by estrogen. Horm. Cancer.

[17] Liu J.L., Ma H.P., Lu X.L., Sun S.H., Guo X., Li F.C. (2011). NF-κB induces abnormal centrosome amplification by upregulation of CDK2 in laryngeal squamous cell cancer. Int. J. Oncol..

[18] Fang Y., Lu Y., Zang X., Wu T., Qi X., Pan S., Xu X. (2016). 3D-QSAR and docking studies of flavonoids as potent *Escherichia coli* inhibitors. Sci. Rep..

[19] Abd-El-Aziz A.S., El-Ghezlani E.G., Elaasser M.M., Afifi T.H., Okasha R.M. (2019). First Example of Cationic Cyclopentadienyliron Based Chromene Complexes and Polymers: Synthesis, Characterization, and Biological Application. J. Inorg. Organomet. Polym..

[20] Okasha R.M., Alblewi F.F., Afifi T.H., Naqvi A., Fouda A.M., Al-Dies A.M., El-Agrody A.M. (2017). Design of new benzo[*h*]chromene derivatives: Antitumor activities and structure-activity relationships of the 2,3-positions and fused rings at the 2,3-positions. Molecules.

[21] Okasha R.M., Alsehli M., Ihmaid S., Althagfan S.S., El-Gaby M.S.A., Ahmed H.E.A., Afifi T.H. (2019). First example of Azo-Sulfa conjugated chromene moieties: Synthesis, characterization, antimicrobial assessment, docking simulation as potent class I histone deacetylase inhibitors and antitumor agents. Bioorg. Chem..

[22] Ahmed H.E.A., El-Nassag M.A.A., Hassan A.H., Okasha R.M., Ihmaid S., Fouda A.M., Afifi T.H., Aljuhani A., El-Agrody A.M. (2018). Introducing novel potent anticancer agents of 1*H*-benzo[*f*]chromene scaffolds, targeting c-Src kinase enzyme with MDA-MB-231 cell line anti-invasion effect. J. Enzyme Inhib. Med. Chem..

[23] Gjini E., Brito P.H. (2016). Integrating Antimicrobial Therapy with Host Immunity to Fight Drug-Resistant Infections: Classical vs. Adaptive Treatment. PLoS Comput. Biol..

[24] Kobayashi M., Kinjo T., Koseki Y., Bourne C.R., Barrow W.W., Aoki S. (2014). Identification of novel potential antibiotics against Staphylococcus using structure-based drug screening targeting dihydrofolate reductase. J. Chem. Inf. Model..

[25] Khalafi-Nezhad A., Soltani Rad M.N., Mohabatkar H., Asrari Z., Hemmateenejad B. (2005). Design, synthesis, antibacterial and QSAR studies of benzimidazole and imidazole chloroaryloxyalkyl derivatives. Bioorg. Med. Chem..

[26] Mandalari G., Bennett R.N., Bisignano G., Trombetta D., Saija A., Faulds C.B., Gasson M.J., Narbad A. (2007). Antimicrobial activity of flavonoids extracted from bergamot (*Citrus bergamia* Risso) peel, a byproduct of the essential oil industry. J. Appl. Microbiol..

[27] Miceli N., Trovato A., Dugo P., Cacciola F., Donato P., Marino A., Bellinghieri V., La Barbera T.M., Guvenc A., Taviano M.F. (2009). Comparative analysis of flavonoid profile, antioxidant and antimicrobial activity of the berries of *Juniperus communis* L. var. communis and *Juniperus communis* L. var. saxatilis Pall. from Turkey. J. Agric. Food Chem..

[28] Orhan D.D., Özçelik B., Özgen S., Ergun F. (2010). Antibacterial, antifungal, and antiviral activities of some flavonoids. Microbiol. Res..

[29] Cushnie T.P., Lamb A.J. (2005). Antimicrobial activity of flavonoids. Int. J. Antimicrob. Agents.

[30] Ventura T.L., Calixto S.D., de Azevedo Abrahim-Vieira B., de Souza A.M., Mello M.V., Rodrigues C.R., Soter de Mariz e Miranda L., Alves de Souza R.O., Leal I.C., Lasunskaia E.B. (2015). Antimycobacterial and anti-inflammatory activities of substituted chalcones focusing on an anti-tuberculosis dual treatment approach. Molecules.

[31] Alcaraz L.E., Blanco S.E., Puig O.N., Tomas F., Ferretti F.H. (2000). Antibacterial activity of flavonoids against methicillin-resistant *Staphylococcus aureus* strains. J. Theor. Biol..

[32] An J., Zuo G.Y., Hao X.Y., Wang G.C., Li Z.S. (2011). Antibacterial and synergy of a flavanonol rhamnoside with antibiotics against clinical isolates of methicillin-resistant *Staphylococcus aureus* (MRSA). Phytomedicine.

[33] Mercader A.G., Pomilio A.P. (2012). 2D- and 3D-QSAR Studies of Flavonoids, Biflavones and Chalcones: Antiviral, Antibacterial, Antifungal, and Antimycobacterial Activities. Anti-Infective Agents.

[34] Forbes A.M., Lin H., Meadows G.G., Meier G.P. (2014). Synthesis and anticancer activity of new flavonoid analogs and inconsistencies in assays related to proliferation and viability measurements. Int. J. Oncol..

[35] Mohamed G.A., Ibrahim S.R., Elkhyat E., Ross S.A., Sayed H.M., El-Moghazy S.A., El-Shanawany M.A. (2015). Blepharisides A and B, new flavonol glycosides from *Blepharis ciliaris* growing in Saudi Arabia. Phytochem. Lett..

[36] Rana A.C., Gulliya B. (2019). Chemistry and Pharmacology of Flavonoids—A Review. Indian. J. Pharm. Educ..

[37] Manach C., Scalbert A., Morand C., Remesy C., Jimenez L. (2004). Polyphenols: Food sources and bioavailability. Am. J. Clin. Nutr..

[38] Agullo G., Gamet-Payrastre L., Manenti S., Viala C., Remesy C., Chap H., Payrastre B. (1997). Relationship between flavonoid structure and inhibition of phosphatidylinositol 3-kinase: A comparison with tyrosine kinase and protein kinase C inhibition. Biochem. Pharmacol..

[39] Haddad A.Q., Venkateswaran V., Viswanathan L., Teahan S.J., Fleshner N.E., Klotz L.H. (2006). Novel antiproliferative flavonoids induce cell cycle arrest in human prostate cancer cell lines. Prostate Cancer Prostatic Dis..

[40] Francisco A. (1995). The Flavonoids- Advances in Research since 1986. Phytochem. Analysis.

[41] Goto H., Terao Y., Akai S. (2009). Synthesis of Various Kinds of Isoflavones, Isoflavanes, and Biphenyl-Ketones and Their 1,1-Diphenyl-2-picrylhydrazyl Radical-Scavenging Activities. Chem. Pharm. Bull..

[42] Woo Y., Shin S.Y., Hyun J., Lee S.D., Lee Y.H., Lim Y. (2012). Flavanones inhibit the clonogenicity of HCT116 cololectal cancer cells. Int. J. Mol. Med..

[43] Szkudelska K., Nogowski L., Nowicka E., Szkudelski T. (2007). In vivo metabolic effects of naringenin in the ethanol consuming rat and the effect of naringenin on adipocytes in vitro. J. Anim. Physiol. Anim. Nutr..

[44] Lee S., Shin S.Y., Lee Y., Park Y., Kim B.G., Ahn J.H., Chong Y., Lee Y.H., Lim Y. (2011). Rhamnetin production based on the rational design of the poplar O-methyltransferase enzyme and its biological activities. Bioorg. Med. Chem. Lett..

[45] Kretzschmar G., Vollmer G., Schwab P., Tischer S., Metz P., Zierau O. (2007). Effects of the chemically synthesized flavanone 7-(O-prenyl)naringenin-4’-acetate on the estrogen signaling pathway in vivo and in vitro. J. Steroid. Biochem..

[46] Voskressensky L.G., Festa A.A., Varlamov A.V. (2014). Domino reactions based on Knoevenagel condensation in the synthesis of heterocyclic compounds. Recent advances. Tetrahedron.

[47] Lipinski C.A., Lombardo F., Dominy B.W., Feeney P.J. (2001). Experimental and computational approaches to estimate solubility and permeability in drug discovery and development settings. Adv. Drug. Del. Rev..

[48] Ghose A.K., Viswanadhan V.N., Wendoloski J.J. (1998). Prediction of hydrophobic (lipophilic) properties of small organic molecules using fragmental methods: An analysis of ALOGP and CLOGP methods. J. Phys. Chem. A.

[49] Veber D.F., Johnson S.R., Cheng H.Y., Smith B.R., Ward K.W., Kopple K.D. (2002). Molecular properties that influence the oral bioavailability of drug candidates. J. Med. Chem..

[50] Egan W.J., Lauri G. (2002). Prediction of intestinal permeability. Adv. Drug Del. Rev..

[51] Muegge I., Heald S.L., Brittelli D. (2001). Simple selection criteria for drug-like chemical matter. J. Med. Chem..

[52] Vanden-Berghe D.A., Vlientinck A.J., Hostettmann K. (1991). Methods in Plant Biochemistry VI. Assays for Bioactivity.

[53] Atta-ur-Rahman M.I.C., Thomsen W.J. (2001). Bioassay Techniques for Drug Development Bioassay Techniques for Drug Development.

[54] Smania J.A., Monache F.D., Smania E.F.A., Cuneo R.S. (1999). Antibacterial Activity of Steroidal Compounds Isolated from *Ganoderma applanatum* (Pers.) Pat. (Aphyllophoromycetideae) Fruit Body. Int. J. Med. Mushrooms.

[55] Mosmann T. (1983). Rapid colorimetric assay for cellular growth and survival: Application to proliferation and cytotoxicity assays. J. Immunol. Methods..

[56] Alley M.C., Scudiero D.A., Monks A., Hursey M.L., Czerwinski M.J., Fine D.L., Abbott B.J., Mayo J.G., Shoemaker R.H., Boyd M.R. (1988). Feasibility of drug screening with panels of human tumor cell lines using a microculture tetrazolium assay. Cancer Res..

[57] (2012). Molecular Operating Environment (MOE) Chemical Computing Group, Quebec, Canada. http://www.chemcomp.com.

[58] Camargo A.J., Honório K.M., Mercadante R., Molfetta F.A., Alves C.N., Da Silva A.B.F. (2003). A study of neolignan compounds with biological activity against paracoccidoiides brasiliensis by using quantum chemical and chemo metric methods. J. Braz. Chem. Soc..

[59] Abd-El-Aziz A.S., Alsaggaf A.T., Okasha R.M., Ahmed H.E.A., Bissessur R., Abdelghani A.A., Afifi T.H. (2016). Antimicrobial and Antitumor Screening of Fluorescent 5,7-Dihydroxy-4-Propyl-2*H*-Chromen-2-One Derivatives with Docking Studies. Chem. Select.

[60] Hutanu C.A., Zaharia M., Pintilie O. (2013). Quenching of Tryptophan Fluorescence in the Presence of 2,4-DNP, 2,6-DNP, 2,4-DNA and DNOC and Their Mechanism of Toxicity. Molecules.

[61] Klancnik A., Piskernik S., Jersek B., Mozina S.S. (2010). Evaluation of diffusion and dilution methods to determine the antibacterial activity of plant extracts. J. Microbiol. Meth..

[62] Gangadevi V., Muthumary J. (2007). Preliminary studies on cytotoxic effect of fungal taxol on cancer cell lines. Afr. J. Biotechnol..

[63] Schüttelkopf A.W., Aalten D.M. (2004). PRODRG: A tool for high-throughput crystallography of protein-ligand complexes. Acta. Cryst..

[64] Morris G.M., Goodsell D.S., Halliday R.S., Huey R., Hart W.E., Belew K.R., Olson A.J. (1998). Automated docking using a lamarckian genetic algorithm and empirical binding free energy function. J. Comput. Chem..

